# Genome-Wide Association Study for Incident Myocardial Infarction and Coronary Heart Disease in Prospective Cohort Studies: The CHARGE Consortium

**DOI:** 10.1371/journal.pone.0144997

**Published:** 2016-03-07

**Authors:** Abbas Dehghan, Joshua C. Bis, Charles C. White, Albert Vernon Smith, Alanna C. Morrison, L. Adrienne Cupples, Stella Trompet, Daniel I. Chasman, Thomas Lumley, Uwe Völker, Brendan M. Buckley, Jingzhong Ding, Majken K. Jensen, Aaron R. Folsom, Stephen B. Kritchevsky, Cynthia J. Girman, Ian Ford, Marcus Dörr, Veikko Salomaa, André G. Uitterlinden, Gudny Eiriksdottir, Ramachandran S. Vasan, Nora Franceschini, Cara L. Carty, Jarmo Virtamo, Serkalem Demissie, Philippe Amouyel, Dominique Arveiler, Susan R. Heckbert, Jean Ferrières, Pierre Ducimetière, Nicholas L. Smith, Ying A. Wang, David S. Siscovick, Kenneth M. Rice, Per-Gunnar Wiklund, Kent D. Taylor, Alun Evans, Frank Kee, Jerome I. Rotter, Juha Karvanen, Kari Kuulasmaa, Gerardo Heiss, Peter Kraft, Lenore J. Launer, Albert Hofman, Marcello R. P. Markus, Lynda M. Rose, Kaisa Silander, Peter Wagner, Emelia J. Benjamin, Kurt Lohman, David J. Stott, Fernando Rivadeneira, Tamara B. Harris, Daniel Levy, Yongmei Liu, Eric B. Rimm, J. Wouter Jukema, Henry Völzke, Paul M. Ridker, Stefan Blankenberg, Oscar H. Franco, Vilmundur Gudnason, Bruce M. Psaty, Eric Boerwinkle, Christopher J. O'Donnell

**Affiliations:** 1 Department of Epidemiology, Erasmus University Medical Center, Rotterdam, The Netherlands; 2 Cardiovascular Health Research Unit, Department of Medicine, University of Washington, Seattle, Washington, United States of America; 3 Department of Biostatistics, Boston University School of Public Health, Boston, MA, United States of America; 4 Icelandic Heart Association, Kopavogur, Iceland; 5 University of Iceland, Reykjavik, Iceland; 6 Human Genetics Center, and Division of Epidemiology, Human Genetics, and Environmental Sciences, University of Texas Health Science Center at Houston, Houston, TX, United States of America; 7 Boston University’s and National Heart Lung and Blood Institute’s Framingham Heart Study, Framingham, MA, United States of America; 8 Department of Cardiology, Leiden University Medical Center, Leiden, The Netherlands; 9 Department of Gerontology and Geriatrics, Leiden University Medical Center, Leiden, The Netherlands; 10 Division of Preventive Medicine, Brigham and Women's Hospital and Harvard Medical School, Boston, Massachusetts, United States of America; 11 Department of Biostatistics, University of Washington, Seattle, WA, United States of America; 12 Department of Statistics, University of Auckland, Auckland, New Zealand; 13 Interfaculty Institute for Genetics and Functional Genomics, University Medicine Greifswald, Greifswald, Germany; 14 DZHK (German Center for Cardiovascular Research), partner site, Greifswald, Germany; 15 Department of Pharmacology and Therapeutics, University College, Cork, Ireland; 16 Department of Internal Medicine, Division of Geriatrics, Wake Forest University, Winston-Salem, North Carolina, United States of America; 17 Department of Nutrition, Harvard School of Public Health, Boston, MA, United States of America; 18 Channing Division of Network Medicine, Harvard Medical School, Boston, MA, United States of America; 19 Division of Epidemiology & Community Health, School of Public Health, University of Minnesota, Minneapolis, United States of America; 20 Sticht Center on Aging, Wake Forest School of Medicine, Winston-Salem, NC, United States of America; 21 Department of Epidemiology, Gillings School of Global Public Health, University of North Carolina, Chapel Hill, NC, United States of America; 22 Department of Epidemiology, Merck Research Laboratories, Merck Sharp & Dohme Corp., Whitehouse Station, NJ, United States of America; 23 Robertson Centre for Biostatistics, University of Glasgow, Glasgow, United Kingdom; 24 Department of Internal Medicine B, University Medicine Greifswald, Greifswald, Germany; 25 National Institute for Health and Welfare, Helsinki, Finland; 26 Department of Internal Medicine, Erasmus University Medical Center, Rotterdam, The Netherlands; 27 Department of Epidemiology, Boston University School of Public Health, Boston, MA, United States of America; 28 Department of Medicine, Boston University School of Medicine, Boston, MA, United States of America; 29 Department of Preventive Medicine, Boston University School of Medicine, Boston, MA, United States of America; 30 Section of Cardiovascular Medicine, Department of Medicine, Boston University School of Medicine, Boston, MA, United States of America; 31 Public Health Sciences, Fred Hutchinson Cancer Research Center, Seattle, WA, United States of America; 32 Department of Epidemiology and Public Health, Pasteur Institute of Lille, Lille, France; 33 Department of Epidemiology and Public Health, EA 3430, University of Strasbourg, Strasbourg, France; 34 Department of Epidemiology, University of Washington, Seattle, WA, United States of America; 35 Group Health Research Institute, Group Health Cooperative, Seattle, United States of America; 36 Departments of Cardiology and Epidemiology, Toulouse University Hospital, Toulouse, France; 37 National Institute of Health and Medical Research (U258), Paris, France; 38 Seattle Epidemiologic Research and Information Center of the Department of Veterans Affairs Office of Research and Development, Seattle, WA, United States of America; 39 Novartis Institutes for Biomedical Research, 250 Massachusetts Avenue, Cambridge, MA, United States of America; 40 The New York Academy of Medicine, New York, NY, United States of America; 41 Department of Medicine, Umeå University Hospital, Umeå, Sweden; 42 Institute for Translational Genomics and Population Sciences, Los Angeles Biomedical Research Institute, Torrance, CA, United States of America; 43 Department of Pediatrics, Harbor-UCLA Medical Center, Torrance, CA, United States of America; 44 UKCRC Centre of Excellence for Public Health Research (Northern Ireland), Queen’s University of Belfast, Belfast, United Kingdom; 45 Department of Mathematics and Statistics, University of Jyväskylä, Jyväskylä, Finland; 46 Department of Epidemiology, Harvard School of Public Health, Boston, MA, United States of America; 47 Laboratory of Epidemiology, Demography, and Biometry, National Institute on Aging, National Institutes of Health, Bethesda, MD, United States of America; 48 Institute for Community Medicine, University Medicine Greifswald, Greifswald, Germany; 49 Institute for Molecular Medicine FIMM, University of Helsinki, Helsinki, Finland; 50 Department of Epidemiology & Prevention, Public Health Sciences, Wake Forest School of Medicine, Winston-Salem, NC, 27157, United States of America; 51 Institute of Cardiovascular and Medical Sciences, Faculty of Medicine, University of Glasgow, Glasgow, United Kingdom; 52 Netherlands Genomics Initiative (NGI)-sponsored Netherlands Consortium for Healthy Aging (NCHA), Leiden, The Netherlands; 53 Durrer Center for Cardiogenetic Research, Amsterdam, The Netherlands; 54 Interuniversity Cardiology Institute of the Netherlands, Utrecht, The Netherlands; 55 Department of General and Interventional Cardiology, University Heart Center Hamburg-Eppendorf, Hamburg, Germany; 56 Department of Health Services, University of Washington, Seattle, WA, United States of America; 57 Department of Medicine, Baylor College of Medicine, Houston, Texas, United States of America; 58 Division of Intramural Research, National Heart, Lung and Blood Institute, Bethesda, MD, United States of America; 59 Cardiology Section, Department of Medicine, Boston Veteran’s Administration Healthcare, Boston, MA, United States of America; Universite de Montreal, CANADA

## Abstract

**Background:**

Data are limited on genome-wide association studies (GWAS) for incident coronary heart disease (CHD). Moreover, it is not known whether genetic variants identified to date also associate with risk of CHD in a prospective setting.

**Methods:**

We performed a two-stage GWAS analysis of incident myocardial infarction (MI) and CHD in a total of 64,297 individuals (including 3898 MI cases, 5465 CHD cases). SNPs that passed an arbitrary threshold of 5×10^−6^ in Stage I were taken to Stage II for further discovery. Furthermore, in an analysis of prognosis, we studied whether known SNPs from former GWAS were associated with total mortality in individuals who experienced MI during follow-up.

**Results:**

In Stage I 15 loci passed the threshold of 5×10^−6^; 8 loci for MI and 8 loci for CHD, for which one locus overlapped and none were reported in previous GWAS meta-analyses. We took 60 SNPs representing these 15 loci to Stage II of discovery. Four SNPs near *QKI* showed nominally significant association with MI (p-value<8.8×10^−3^) and three exceeded the genome-wide significance threshold when Stage I and Stage II results were combined (top SNP rs6941513: p = 6.2×10^−9^). Despite excellent power, the 9p21 locus SNP (rs1333049) was only modestly associated with MI (HR = 1.09, p-value = 0.02) and marginally with CHD (HR = 1.06, p-value = 0.08). Among an inception cohort of those who experienced MI during follow-up, the risk allele of rs1333049 was associated with a decreased risk of subsequent mortality (HR = 0.90, p-value = 3.2×10^−3^).

**Conclusions:**

*QKI* represents a novel locus that may serve as a predictor of incident CHD in prospective studies. The association of the 9p21 locus both with increased risk of first myocardial infarction and longer survival after MI highlights the importance of study design in investigating genetic determinants of complex disorders.

## Introduction

There is strong and consistent evidence that coronary heart disease (CHD) is highly heritable and is influenced by a wide range of genetic factors [1, 2]. Recently genome-wide association studies (GWAS) identified common genetic variants involved in cardiovascular disease and its risk factors [3]. The loci reported by the latest and largest GWAS altogether explain around 10% of CHD heritability [4].

To date, GWAS for CHD have been conducted mostly in cross-sectional case-control setting, and this design, which uses prevalent cases, typically oversamples those with long post-event survival times. Although such a design often makes it possible to collect information from a large number of patients, this approach may incorrectly identify factors that are associated with a high or low case-fatality rate. For instance, a factor associated with a low case-fatality will be enriched among surviving cases and may appear to increase the risk of disease when prevalent cases are compared with controls. This bias is known as incidence-prevalence (Neyman) bias [5, 6]. One major advantage of studying incident cases rather than prevalent cases is that incident cases properly represent the fatal cases and persons with only brief post-event survival. To date the strong and reliable evidence for identifying and assessing factors such as LDL-cholesterol and systolic blood pressure that predict future clinical disease are provided by well-designed population-based, prospective cohort studies that collect large number of incident cases [7].

Here we aimed to study genetic variants that affect the incidence of myocardial infarction (MI) and CHD in prospective, population-based cohorts and whether the genetic variants identified to date are also associated with risk of CHD in a prospective setting. Moreover, we investigated whether the known genetic variants are associated with total-mortality after MI. To this end we used the data from the Cohorts for Heart and Aging Research in Genome Epidemiology (CHARGE) Consortium [8] and collaborating prospective studies.

## Methods

### Study Population

We performed our study in two stages. Stage I studies comprised participants from five prospective cohort studies that form the CHARGE consortium [8]: the Age, Gene Environment Susceptibility Reykjavik Study (AGES) [9]; the Atherosclerosis Risk in Communities (ARIC) Study [10]; the Cardiovascular Health Study (CHS) [11]; the Framingham Heart Study (FHS) [12]; and the Rotterdam Study (RS) [13, 14]. Stage II comprised individuals from: The Health, Aging, and Body Composition (Health ABC) Study; The Health Professionals Follow-Up Study (HPFS); The Nurses’ Health Study (NHS); PROSPER/PHASE Study; the Study of Health in Pomerania (SHIP); The Women’s Genome Health Study (WGHS); the MOnica Risk, Genetics, Archiving and Monograph (MORGAM) Study comprising the Alpha-Tocopherol, Beta-Carotene (ATBC) Study; The FINRISK Study; The PRIME Study (including the PRIME cohorts of Belfast, Lille, Strasbourg and Toulouse); The Northern Sweden Study. Participants in Stages I and II were of European ancestry. Participants with a history of MI or CHD at baseline were excluded. All studies had protocols approved by local institutional review boards. Participants provided written informed consent and gave permission to use their DNA for research purposes. The Supplementary Document provides details about the design and characteristics for these studies.

### Case Definitions for MI and CHD

The definitions of incident MI were consistent among the participating studies, including both fatal and non-fatal MI. CHD included fatal or non-fatal MI, and in most studies fatal CHD or sudden death. The definition of MI and CHD for each cohort study is summarized in S1 Table and S2 Table.

### Statistical Analysis

The date of entry to the analysis was the date of cohort entry (AGES, ARIC, CHS, RS) or DNA collection (FHS). Within each study, Cox proportional hazards regression models were used to test the association between each SNP and time to incident MI or CHD, while adjusting for sex and baseline age. FHS adjusted for familial correlation by clustering on pedigree. Analyses in CHS and ARIC were adjusted for study site and in FHS, for generation and additionally for ancestry using principal components [15]. The censor date was the time of MI or CHD diagnosis, the time of death, last date of contact, or at the end of follow-up, whichever came first. For each SNP, additive genetic models were used to estimate the regression coefficient for the hazard ratio (HR) for allele dosage and its respective standard error. For each analysis, a genomic control coefficient (λ) was calculated, which estimated the extent of underlying population structure. Further information on the analysis methods can be found in S3 Table and S4 Table.

Information regarding the genotyping and imputation as well as genotype quality control are found in S5 Table and S6 Table. SNPs with a minor allele frequency of less than 1%, imputation quality less than 0.3 or very large regression coefficients (absolute value larger than 5) were excluded from meta-analysis. Results from individual studies were meta-analyzed for a total of 2,543,842 autosomal SNPs based on Phase 2 HapMap. A fixed effects inverse variance weighted meta-analysis approach was implemented in METAL [16] to combine the regression coefficients and their standard errors, producing a summary regression coefficient and standard error from which a p-value was computed. An arbitrary significance threshold for follow-up in Stage II was set at 5.0×10^−6^. When more than one SNP clustered at a locus, we carried forward four SNPs with smallest p-values in the associated locus for further investigation in Stage II.

In Stage II, three studies provided data both for incident MI and CHD (HABC, MORGAM, and WGHS), two studies provided data only for MI (PROSPER, SHIP), and two others provided data only for CHD (HPFS, NHS). Each Stage II study used the same analytic method as used in Stage I to examine the association of the 60 SNPs with MI or CHD. As in the Stage I meta-analysis, we used inverse-variance weighted fixed effects meta-analysis to evaluate the Stage II results. We applied a Bonferroni correction for 60 SNPs and set 8.3×10^−4^ as the significance threshold. Finally, results from all studies in Stage I and II were combined using inverse-variance weighted fixed effects meta-analysis.

We further studied each of the 46 SNPs reported by the CARDIoGRAMplusC4D Consortium [4], for association with incident events in our meta-analysis of longitudinal cohort studies. Moreover, the SNPs were combined into a weighted genetic risk score using beta estimates from the CARDIoGRAMplusC4D Consortium report [4]. The association of each SNP, as well as the score from the combination of all 46 SNPs, was examined with incident MI and CHD using the results of Stage I meta-analysis.

We applied a Cox proportional hazards model adjusted for age and sex to examine the association of the known SNPs with mortality after MI. Five studies including AGES, ARIC, CHS, FHS and the Rotterdam Study provided data for this analysis and in total 2953 individuals were followed after incidence of MI of which 1828 died. The median follow up time ranged from 2.3 years in AGES to 4.7 years in FHS. The baseline characteristics of the study populations for this analysis are presented in S7 Table. Since this analysis was meant to explore potential reasons for weak association or lack of association with incident MI and CHD, we limited the analysis to three SNPs with more than 80% power in Stage I to study its estimated associations with incident MI and CHD.

## Results

**Fig 1:**
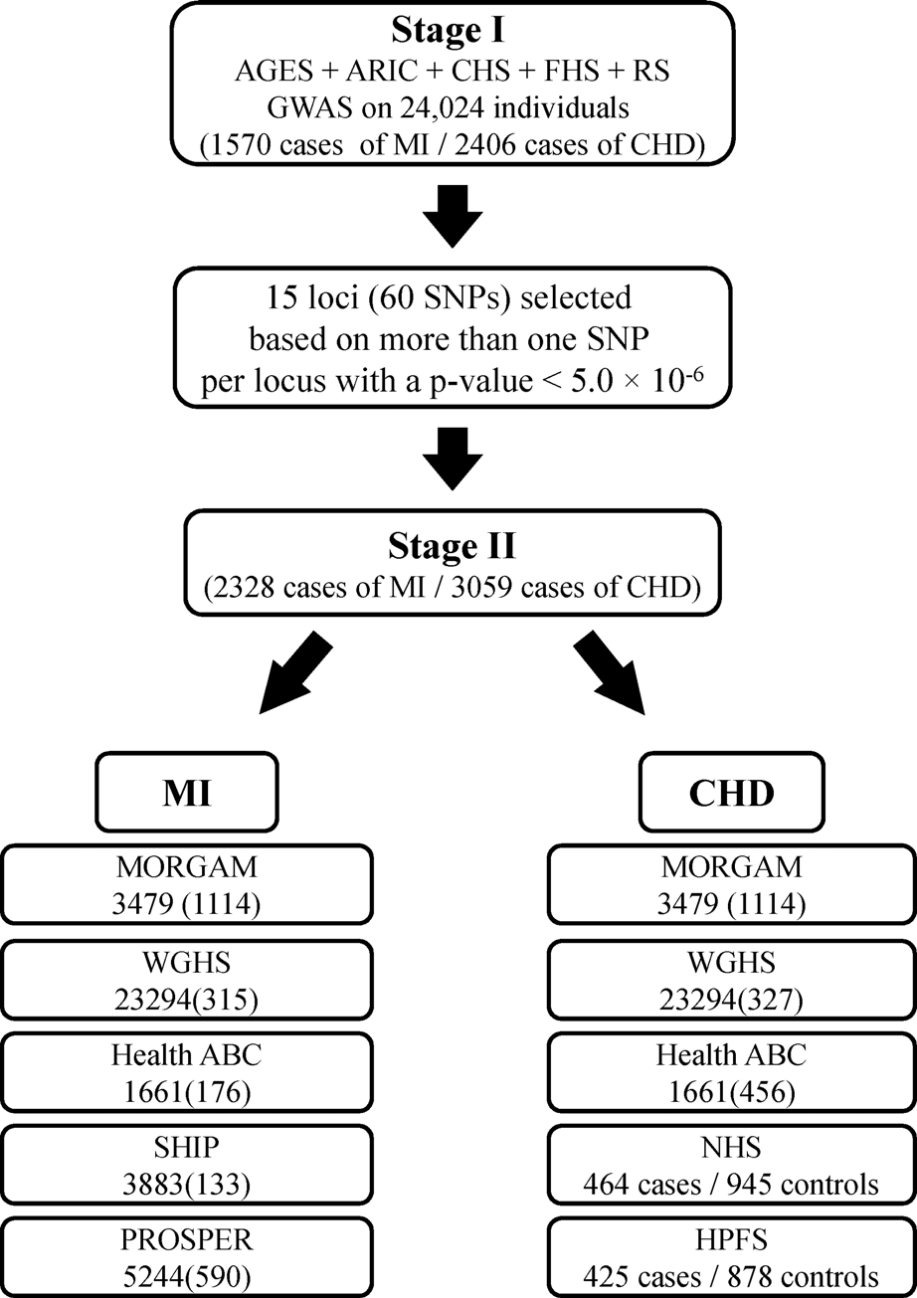
Study design for identification and validation of SNPs associated with MI and CHD.

Fig 1 describes Stage I and Stage II of the study. The Stage I panel included five prospective cohort studies comprising a total of 24,024 participants who were free of MI and CHD at baseline. The average age ± standard deviation ranged from 54.1±5.6 in ARIC to 74.6±5.5 in AGES. More than half of the participants (54.5%) were women. The basic characteristics of the participating studies are shown in Table 1. A total of 1570 incident MI events (6.5%) and 2406 incident CHD events (10.0%) occurred over an average of 8.2 years and 8.1 years of follow-up for MI and CHD, respectively. The average age at the time of MI ranged from 65.2 years in ARIC to 80.8 years in CHS.

**Table 1:** Baseline characteristics of participants included in incident MI/CHD analysis stratified by cohort.

Characteristic	AGES	ARIC	CHS	FHS	RS
Participants, n	3219	7406	3291	4134	5974
Age, years	76.4 (5.5)	54.1 (5.57)	72.3 (5.4)	64.5 (12.8)	69.4 (9.1)
Women, %	58.0	54.7	39.1	56.7	59.4
Hypertension^1^, %	80.6	25.7	52.8	45.3	33.4
Diabetes^2^, %	11.5	7.7	11.8	10.2	10.6
Current smoker^3^, %	12.7	24.8	11.3	14.0	22.4
Total cholesterol, mg/dL	217 (45)	214 (41)	213 (39)	203 (40)	255 (47)
HDL cholesterol, mg/dL	61 (17)	51 (17)	55 (16)	52 (17)	52 (14)
Triglyceride, mg/dL	107 (59)	135 (91)	140 (76)	144 (127)	NA
Body mass index, kg/m^2^	27.1 (4.4)	27.0 (4.9)	26.3 (4.5)	27.7 (5.2)	26.3 (3.7)
Incident MI, N cases	86	486	537	165	296
Mean MI follow-up time	2.7	9.2	12.0	5.5	10.1
Incident CHD, N cases	209	575	660	201	761
Mean CHD follow-up time	2.6	9.1	12.0	5.5	9.9
Incident MI Age, years	79.1 (5.5)	65.17 (6.9)	80.8 (6.2)	75.2 (12.2)	80.6 (10.1)

Numbers in table are Mean (SD) or percentage. AGES = Age, Gene/Environment Study; ARIC = Atherosclerosis Risk in Communities Study; CHS = Cardiovascular Health Study; FHS = Framingham Heart Study; HDL = high density lipoprotein; RS = The Rotterdam Study

1 Hypertension was defined as blood pressure ≥140/90 mmHg or on anti-hypertensive medication

2 Diabetes was defined as fasting blood glucose >125 mg/dL, a random blood glucose of >200 mg/dL, or use of insulin or oral hypoglycemic agents (Rotterdam: diabetes definition: Using anti-diabetic medication or random glucose or oral glucose test more than 200 mg/dl)

3 Current cigarette smoking was defined as self-reported cigarette smoking of at least 1 cigarette per day for a year at any attended exam

The λ coefficient within each cohort was small (≤1.03), suggesting negligible genomic inflation. We combined the results of associations for all SNPs across the five cohorts. S1A Fig and S1B Fig presents the Q-Q plots of combined p-values against the expected p-value distribution for MI and CHD, respectively. The evidence for population admixture was small, both for MI (λ = 1.017) and CHD (λ = 1.022). S2A Fig and S2B Fig illustrates the p-values of the meta-analysis for each of the SNPs across the 22 autosomal chromosomes for MI and CHD, respectively.

In Stage I, 27 SNPs in 8 loci reached our arbitrary threshold of 5×10^−6^ for MI and 29 SNPs in 8 loci reached this threshold for CHD (Table 2). The most significant association with MI was seen for rs6941513 located on chromosome 6 upstream of *QKI* (Hazard Ratio = 1.22 [95% Confidence Interval: 1.13, 1.31], p-value = 2.0×10^−7^). For CHD, rs986080, a SNP located on chromosome 1 between two genes (*SNX7* and *PAP2D*) showed the strongest association (HR = 1.19 [95%CI: 1.12, 1.27], p-value = 6.6×10^−8^).

**Table 2:** Description and association of SNPs of the top loci associated with incident MI and CHD in Stage I.

Phenotype	SNP	Band	Alleles*	HR (95%CI)	P-value	Gene	Location
MI	rs6941513	6q26	G/A	1.22 (1.13–1.31)	2.0×10^−7^	*QKI*	Closest gene
	rs13139636	4q35.1	T/A	1.44 (1.25–1.66)	6.4×10^−7^	*ODZ3*	Intron
	rs217597	7p21.2	T/C	1.26 (1.15–1.38)	1.3×10^−6^	*DGKB*	Intron
	rs9923194	16q24.1	C/T	1.88 (1.44–2.44)	1.9×10^−6^	*FOXL1*	Closest gene
	rs6504582	17q21.32	A/G	1.20 (1.11–1.30)	2.1×10^−6^	*CALCOCO2*	Closest gene
	rs7591615	2q35	T/C	1.21 (1.12–1.32)	3.3×10^−6^	*BARD1*	Intron
	rs17777478	3q12.1	T/A	1.73 (1.37–2.20)	4.4×10^−6^	*COL8A1*	Intron
	rs2299063	6p22.3	A/C	1.24 (1.13–1.37)	4.8×10^−6^	*ATXN1*	Intron
CHD	rs986080	1p21.3	C/T	1.19 (1.12–1.27)	6.6×10^−8^	*PAP2D*	Intron
	rs16945166	13q31.3	G/A	1.34 (1.20–1.51)	4.7×10^−7^	*GPC5*	Closest gene
	rs10740220	10q21.3	G/T	1.20 (1.11–1.29)	1.0×10^−6^	*CTNNA3*	Intron
	rs10922855	1p22.2	T/G	1.22 (1.12–1.33)	1.9×10^−6^	*BARHL2*	Closest gene
	rs7794677	7p12.3	T/C	1.20 (1.11–1.29)	2.3×10^−6^	*IGFBP3*	Closest gene
	rs2916260	6p21.2	T/C	1.21 (1.12–1.31)	2.8×10^−6^	*LRFN2*	Intron
	rs3812189	6p22.3	C/T	1.21 (1.12–1.31)	3.1×10^−6^	*ATXN1*	Intron
	rs356228	4q22.1	G/C	1.16 (1.09–1.23)	3.2×10^−6^	*SNCA*	Closest gene

*Coded/non-coded allele

HR = Hazard Ratio; CI = Confidence Interval

**Fig 2:**
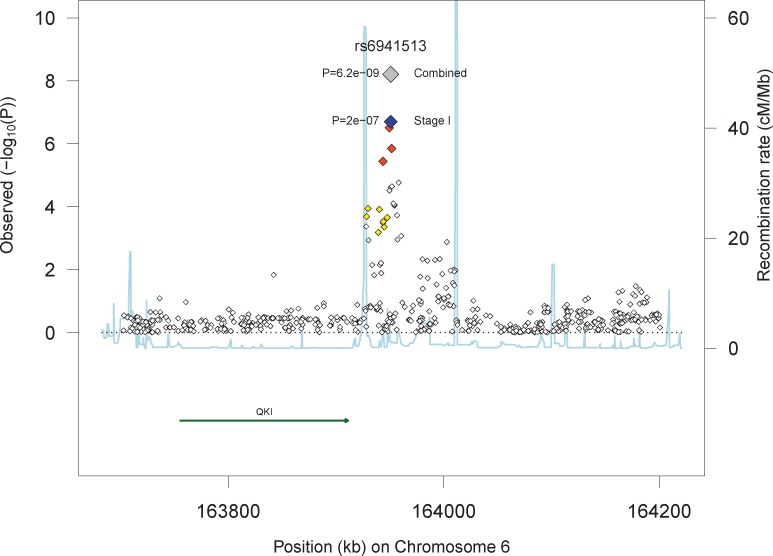
Regional plots for the association of SNPs with MI in the region of *QKI*.

In Stage II, we sought additional evidence for associations in eight loci for MI (*QKI*, *ODZ3*, *DGKB*, *FOXL1*, *CALCOCO2*, *BARD1*, *COL8A1*, *ATXN1*) and eight loci for CHD (*PAP2D*, *GPC5*, *CTNNA3*, *BARHL2*, *IGFBP3*, *LRFN2*, *ATXN1*, *SNCA*) using four SNPs per locus, for a total of 60 SNPs in 15 loci (*ATXN1* was associated with both MI and CHD). Baseline characteristics of the participants of Stages II are shown in S8 and S9 Tables. The results for all 60 SNPs are presented in S10 Table and S11 Table, for MI and CHD, respectively. None of the SNPs passed the Bonferroni adjusted threshold of 8.3×10^−4^. The results for the best association in each locus are shown in Table 3. Four SNPs located upstream of *QKI* showed nominal evidence in Stage II for association with MI. The analysis of the combined Stage I and Stage II yielded genome-wide significant associations for three SNPs close to *QKI*, (rs6941513: HR = 1.21 [95%CI: 1.13, 1.28], p-value = 6.2×10^−9^). Fig 2 presents the linkage disequilibrium (LD) and p-values of regional markers for this locus. We tested for evidence of replication of this association in 8201 African American individuals including 546 incident cases from the PAGE Study [17], however, rs6941513 was not significantly associated with risk of MI in this population (p = 0.49).

**Table 3:** Description and association of top SNPs with incident MI and CHD in Stage II and their combined results with Stage I.

Phenotype	SNPID	Alleles*	Stage II		Combined		Closest Gene
			HR (95%CI)	P-value	HR (95%CI)	P-value	
**MI**	rs6941513	G/A	1.16 (1.04–1.31)	8.8×10^−3^	1.2 (1.13–1.28)	6.2×10^−9^	*QKI*
	rs7692395	T/G	1.03 (0.75–1.40)	0.86	0.69 (0.59–0.79)	2.5×10^−7^	*ODZ3*
	rs4721377	T/G	0.98 (0.87–1.10)	0.73	1.26 (1.14–1.38)	1.9×10^−6^	*DGKB*
	rs9923194	C/T	1.04 (0.69–1.58)	0.84	1.58 (1.27–1.97)	4.3×10^−5^	*FOXL1*
	rs6504582	A/G	1.03 (0.91–1.15)	0.65	1.15 (1.08–1.22)	1.8×10^−5^	*CALCOCO2*
	rs7591615	T/C	0.89 (0.78–1.02)	0.09	1.11 (1.03–1.19)	3.8×10^−3^	*BARD1*
	rs17777478	T/A	1.15 (0.80–1.64)	0.45	1.54 (1.26–1.88)	1.6×10^−5^	*COL8A1*
	rs2299063	A/C	0.93 (0.81–1.07)	0.29	1.13 (1.05–1.23)	1.6×10^−3^	*ATXN1*
**CHD**	rs986080	C/T	0.97 (0.91–1.04)	0.39	0.97 (0.91–1.04)	1.1×10^−3^	*PAP2D*
	rs16945166	G/A	1.02 (0.92–1.14)	0.69	1.02 (0.92–1.14)	2.2×10^−4^	*GPC5*
	rs10509258	C/T	1.02 (0.96–1.09)	0.50	1.02 (0.96–1.09)	3.0×10^−4^	*CTNNA3*
	rs12031583	G/A	0.89 (0.79–1.00)	0.05	0.89 (0.79–1.00)	7.8×10^−3^	*BARHL2*
	rs1551837	A/G	1.03 (0.94–1.12)	0.58	1.03 (0.94–1.12)	2.3×10^−4^	*IGFBP3*
	rs6925172	C/T	1.12 (1.01–1.25)	0.04	1.12 (1.01–1.25)	3.3×10^−6^	*LRFN2*
	rs9297015	T/A	0.93 (0.86–1.01)	0.07	0.93 (0.86–1.01)	0.12	*ATXN1*
	rs356228	G/C	1.02 (0.96–1.09)	0.50	1.02 (0.96–1.09)	1.7×10^−4^	*SNCA*

*Coded/non-coded allele

Chr. = Chromosome; HR = Hazard Ratio; CI = Confidence Interval

We sought evidence for the association of 46 SNPs recently reported in the largest GWAS to date for coronary artery disease [4] with the incidence of MI and CHD (Table 4). Despite excellent power, we found only modest evidence for replication of the association with 9p21 locus (*CDKN2A/B*), the most established finding from previous cross-sectional case-control GWAS. The most replicated SNP at 9p21 locus, rs1333049, was nominally associated with MI (HR: 1.09 [95%CI: 1.01, 1.18], p-value = 0.02) and marginally with CHD (HR = 1.06 [95%CI: 0.99, 1.13], p-value = 0.08). The most significant association with MI was found for rs15563, a SNP in *UBE2Z* (HR: 1.12 [95%CI: 1.04, 1.20], p-value = 1.9×10^−3^) and the most significant association with CHD was found for rs10947789, a SNP within the *KCNK5* locus (HR: 1.13 [95%CI: 1.05, 1.22], p-value = 5.6×10^−4^). We found nominally significant associations (p<0.05) with SNPs annotated to *CDKN2A/B* for MI, *LIPA* for CHD and *COL4A2*, *TCF21*, *PDGFD*, *KCNK5*, *VAMP8*, *MRAS*, *UBE2Z* and *TCF21* for both MI and CHD (Table 4). A weighted genetic risk score composed of these 46 SNPs was associated with MI (p-value = 1.3×10^−3^) and CHD (p-value = 1.2×10^−3^) in the Stage I meta-analysis.

**Table 4:** Association of the known SNPs for coronary artery disease with incident MI and CHD in Stage I.

SNP	Chr.	Freq.	Alleles*	Reported GWAS		GWAS on Incident MI			GWAS on Incident CHD			Gene
				OR	P-value	Power	HR (95% CI)	P-value	Power	HR (95% CI)	P-value	
rs3217992	9	0.38	A/G	1.16	7.8×10^−57^	0.98	1.07 (0.99–1.15)	0.10	0.99	1.04 (0.98–1.11)	0.21	*CDKN2A/B*
rs1333049	9	0.47	C/G	1.23	1.4×10^−52^	0.99	1.09 (1.01–1.18)	0.02	0.99	1.06 (0.99–1.13)	0.08	*CDKN2A/B*
rs602633	1	0.77	C/A	1.12	1.5×10^−25^	0.73	1.08 (0.99–1.17)	0.10	0.89	1.05 (0.97–1.12)	0.21	*PSRC1*
rs9369640	6	0.65	A/C	1.09	7.5×10^−22^	0.62	1.05 (0.98–1.13)	0.17	0.78	1.03 (0.97–1.10)	0.27	*PHACTR1*
rs11556924	7	0.65	C/T	1.09	6.7×10^−17^	0.62	1.05 (0.97–1.14)	0.26	0.78	1.06 (0.99–1.14)	0.07	*ZC3HC1*
rs9982601	21	0.13	T/C	1.13	7.7×10^−17^	0.63	0.98 (0.88–1.09)	0.68	0.79	1.01 (0.93–1.10)	0.81	*MRPS6*
rs6725887	2	0.11	C/T	1.12	1.2×10^−15^	0.51	1.06 (0.95–1.18)	0.29	0.67	0.99 (0.90–1.08)	0.78	*WDR12*
rs1122608	19	0.76	G/T	1.10	6.3×10^−14^	0.62	1.07 (0.98–1.16)	0.13	0.77	1.04 (0.97–1.12)	0.23	*SMARCA4*
rs12190287	6	0.59	C/G	1.07	4.9×10^−13^	0.45	1.13 (1.04–1.23)	4.4×10^−3^	0.60	1.09 (1.01–1.16)	0.02	*TCF21*
rs7173743	15	0.58	T/C	1.07	6.7×10^−13^	0.45	1.05 (0.97–1.13)	0.23	0.60	1.04 (0.98–1.10)	0.23	*MORF4L1*
rs17114036	1	0.91	A/G	1.11	5.8×10^−12^	0.39	1.12 (0.97–1.28)	0.11	0.52	1.06 (0.95–1.19)	0.27	*PPAP2B*
rs9515203	13	0.74	T/C	1.08	5.9×10^−12^	0.46	1.06 (0.95–1.17)	0.31	0.61	1.07 (0.99–1.17)	0.09	*COL4A2*
rs2505083	10	0.42	C/T	1.06	1.4×10^−11^	0.35	1.05 (0.97–1.13)	0.19	0.47	1.03 (0.97–1.10)	0.36	*KIAA1462*
rs4773144	13	0.42	G/A	1.07	1.4×10^−11^	0.45	1.09 (1.01–1.18)	0.03	0.60	1.09 (1.02–1.16)	0.01	*COL4A2*
rs7692387	4	0.81	G/A	1.06	2.7×10^−11^	0.39	1.00 (0.91–1.09)	0.95	0.52	1.01 (0.93–1.09)	0.83	*GUCY1A3*
rs974819	11	0.29	A/G	1.07	3.6×10^−11^	0.39	0.91 (0.83–0.98)	0.02	0.53	0.93 (0.87–1.00)	0.04	*PDGFD*
rs3184504	12	0.40	T/C	1.07	5.4×10^−11^	0.44	1.03 (0.95–1.11)	0.47	0.60	1.04 (0.98–1.11)	0.22	*SH2B3*
rs2075650	19	0.14	G/A	1.11	5.9×10^−11^	0.53	1.03 (0.91–1.16)	0.67	0.68	1.01 (0.91–1.13)	0.79	*TOMM40*
rs2048327	6	0.35	G/A	1.06	6.9×10^−11^	0.33	1.00 (0.93–1.08)	0.98	0.45	1.01 (0.95–1.08)	0.65	*SLC22A3*
rs9319428	13	0.32	A/G	1.05	7.3×10^−11^	0.32	0.99 (0.91–1.09)	0.91	0.43	1.03 (0.96–1.11)	0.44	*FLT1*
rs17514846	15	0.44	A/C	1.05	9.3×10^−11^	0.45	1.00 (0.93–1.08)	0.94	0.60	1.02 (0.96–1.09)	0.52	*FURIN*
rs1561198	2	0.45	A/G	1.05	1.2×10^−10^	0.35	1.09 (1.01–1.17)	0.02	0.48	1.09 (1.02–1.15)	6.9×10^−3^	*VAMP8*
rs515135	2	0.83	G/A	1.08	2.6×10^−10^	0.29	1.09 (0.98–1.20)	0.10	0.40	1.03 (0.95–1.12)	0.52	*APOB*
rs4845625	1	0.47	T/C	1.04	3.6×10^−10^	0.36	1.01 (0.94–1.08)	0.86	0.48	1.03 (0.97–1.10)	0.28	*IL6R*
rs2895811	14	0.43	C/T	1.06	4.1×10^−10^	0.35	1.03 (0.95–1.11)	0.44	0.48	1.03 (0.96–1.10)	0.39	*KIAA1822*
rs4252120	6	0.73	T/C	1.06	4.9×10^−10^	0.38	0.99 (0.92–1.07)	0.82	0.51	0.99 (0.93–1.06)	0.74	*PLG*
rs273909	5	0.14	C/T	1.09	9.6×10^−10^	0.26	1.04 (0.91–1.17)	0.59	0.35	1.01 (0.91–1.12)	0.84	*SLC22A4*
rs12936587	17	0.59	G/A	1.06	1.2×10^−9^	0.35	1.00 (0.92–1.08)	0.94	0.47	0.97 (0.91–1.04)	0.40	*RAI1*
rs2047009	10	0.48	C/A	1.05	1.6×10^−9^	0.27	1.02 (0.94–1.09)	0.68	0.36	1.03 (0.97–1.1)	0.30	*CXCL12*
rs501120	10	0.83	A/G	1.07	1.8×10^−9^	0.29	1.08 (0.96–1.21)	0.18	0.39	1.00 (0.91–1.09)	0.96	*CXCL12*
rs9818870	3	0.14	T/C	1.07	2.6×10^−9^	0.26	1.10 (1.00–1.21)	0.05	0.35	1.09 (1.01–1.18)	0.02	*MRAS*
rs264	8	0.86	G/A	1.05	2.9×10^−9^	0.53	0.99 (0.89–1.10)	0.83	0.68	1.05 (0.96–1.14)	0.31	*LPL*
rs2281727	17	0.36	C/T	1.05	7.8×10^−9^	0.25	1.07 (0.99–1.15)	0.09	0.34	1.02 (0.96–1.09)	0.50	*SMG6*
rs445925	19	0.9	C/T	1.13	8.8×10^−9^	0.54	1.16 (0.97–1.39)	0.10	0.56	1.05 (0.92–1.19)	0.46	*APOC1*
rs10947789	6	0.76	T/C	1.06	9.8×10^−9^	0.36	1.12 (1.02–1.22)	0.01	0.48	1.13 (1.05–1.22)	5.6×10^−4^	*KCNK5*
rs579459	9	0.21	C/T	1.07	2.7×10^−8^	0.33	1.06 (0.97–1.17)	0.20	0.45	1.03 (0.96–1.12)	0.39	*ABO*
rs2252641	2	0.46	G/A	1.04	5.3×10^−8^	0.36	1.00 (0.92–1.07)	0.91	0.48	0.97 (0.91–1.03)	0.34	*ZEB2*
rs12413409	10	0.89	G/A	1.10	6.3×10^−8^	0.38	1.05 (0.92–1.20)	0.49	0.52	1.01 (0.90–1.13)	0.91	*CNNM2*
rs9326246	11	0.10	C/G	1.09	1.5×10^−7^	0.51	0.93 (0.79–1.08)	0.32	0.41	0.93 (0.82–1.06)	0.26	*BUD13*
rs11203042	10	0.44	T/C	1.04	6.1×10^−6^	0.19	1.05 (0.98–1.13)	0.15	0.25	1.07 (1.01–1.14)	0.03	*LIPA*
rs15563	17	0.52	C/T	1.04	9.4×10^−6^	0.19	1.12 (1.04–1.20)	1.9×10^−3^	0.25	1.07 (1.01–1.13)	0.03	*UBE2Z*
rs2246833	10	0.38	T/C	1.06	9.5×10^−6^	0.34	1.07 (1.00–1.16)	0.06	0.46	1.06 (1.00–1.13)	0.05	*LIPA*
rs11206510	1	0.84	T/C	1.06	1.8×10^−5^	0.22	1.02 (0.92–1.14)	0.67	0.30	1.01 (0.93–1.10)	0.80	*PCSK9*
rs12205331	6	0.81	C/T	1.04	4.2×10^−5^	0.14	1.03 (0.93–1.13)	0.58	0.17	1.05 (0.97–1.13)	0.24	*ANKS1A*
rs17464857	1	0.87	T/G	1.05	6.1×10^−5^	0.15	1.11 (0.97–1.27)	0.13	0.19	1.09 (0.97–1.22)	0.14	*TAF1A*
rs12539895	7	0.19	A/C	1.08	5.3×10^−4^	0.39	1.03 (0.94–1.12)	0.57	0.52	0.99 (0.92–1.07)	0.87	*GPR22*

Chr. = Chromosome; Freq. = Frequency; OR = Odds Ratio; HR = Hazard Ratio; CI = Confidence Interval

*Coded / Non-coded allele

**Fig 3:**
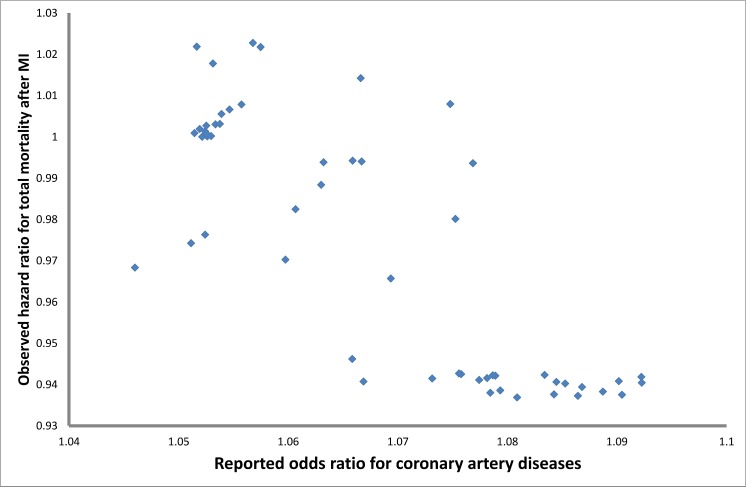
The association of top 79 SNPs with coronary artery disease as reported by CardiogramplusC4D for 9p21 locus and their association with total mortality after MI.

Among individuals who experienced MI during follow-up, the risk allele of rs1333049 was associated with a significantly decreased risk of mortality (HR: 0.90 [95% CI: 0.84, 0.97], p-value = 5.5×10^−3^) (Table 5). In both SNPs at 9p21 locus the “risk allele” from cross-sectional case-control GWAS was associated with longer survival after MI and would have been enriched in surviving prevalent cases. Fig 3 illustrates the inverse association of 78 top SNPs at the 9p21 locus as reported by CARDIoGRAMplusC4D Consortium [4] with survival after MI. We also examined the association of rs6941513 with mortality after MI, however, the association was not significant.

**Table 5:** Association of the known SNPs for coronary artery disease with mortality after MI.

SNP	Closest Gene	Alleles	HR(95%CI)	P-value
rs1333049	*CDKN2A/B*	C/G	0.90 (0.84–0.97)	5.5×10^−3^
rs3217992	*CDKN2A/B*	A/G	0.94 (0.88–1.01)	0.10
rs602633	*PSRC1*	C/A	1.01 (0.93–1.09)	0.93

## Discussion

We performed a GWAS on incident MI and CHD and examined whether the gene variants identified to date are also associated with risk of CHD in a prospective setting. In a two-stage design, involving 37,561 participants with 2,328 cases of incident MI, we identified a novel genome-wide significant locus, *QKI*, associated with incident MI. This finding requires further replication. The results also highlighted the difference between the genes identified in prospective versus cross-sectional case-control studies. The 9p21 locus was associated with both an increased risk of incident MI and, during follow-up post-MI, a decreased risk of total mortality, indicating that genetic variants may operate differently in an alternative setting.

In this two-stage design, we found evidence for MI-associated genetic variants nearby *QKI* (KH domain containing, RNA binding). The combined p-value for three out of four genetic variants that were examined in the region exceeded genome-wide significant threshold. Although these data provide evidence for an association between the *QKI* locus and incident MI, this finding should be confirmed by further studies since these variants attained conventional levels of genome-wide significant p-value only in the combined meta-analysis.

If confirmed, the *QKI* finding may represent a novel pathway in developing CHD. *QKI* is known to be involved in cell cycle regulation, a pathway for which there is emerging evidence for a key role in developing atherosclerotic plaques and cardiovascular disease [18, 19]. A functional study has reported that *QKI* is a central regulator of vascular smooth muscle cell phenotypic plasticity and that intervention in *QKI* activity can improve pathogenic fibro-proliferative responses to vascular injury [20]. Moreover, a recent paper shows that the RNA-binding properties of QKI play a critical role in regulating human monocyte to macrophage differentiation [21]. de Bruin and co-workers identified that the conversion of monocytes to both pro- and anti-inflammatory macrophages with GM-CSF or M-CSF, respectively, markedly increased expression of the QKI, which all were readily detected in CD68+ macrophages of fibrous cap atheromata and atherosclerotic lesions with intraplaque hemorrahage. Furthermore, reduced expression of QKI in monocytes delayed their differentiation into macrophages, perturbed their capacity to become lipid-engorged foam cells, and led to a reduction in monocyte infiltration in atherosclerotic lesions [21]. Altogether we propose that QKI is involved in inflammatory responses to injury and could be a potential thrapeutic target to prevent cardiovascular disease. Further functional investigation is needed to robustly identify mechanisms involved for this locus.

Prior GWAS which included extremely large sample sizes did not report *QKI* though they should have had enough statistical power to detect a locus with such an effect. However rs6941513 was not associated with CAD in the Cardiogram plusC4D GWAS (OR = 1.01, p-value = 0.45). In contrast to former GWAS, we have used a prospective, longitudinal cohort design to examine genetic association with incident cases of MI and CHD. It is possible that the magnitude of the effect with prevalent cases is smaller than with incident cases; thus the locus was not detected by previously published GWAS that primarily use a case-control design.

Although CHD includes MI events by definition, the loci we found for MI and CHD overlapped only for one locus (*ATX1*). One reason could be differences in mechanisms involved in the restrictive diagnosis of MI versus the broader diagnosis of CHD. However, unstable effect estimates and p-values due to lack of statistical power could have contributed to this observation as well.

Despite excellent statistical power, we identified only a modest signal at the 9p21 locus. This locus, initially identified by GWAS, has been validated by numerous studies in different geographic and ethnic subgroups. However, our study is not the first study to report a weak signal or lack of association at this locus. In fact, prominent differences have been observed between cross-sectional case-control versus longitudinal studies. For instance, in a meta-analysis by Chan et al [22], cross-sectional analyses of angiographically defined cases and controls show a strong per allele association with 9p21 (OR: 1.31, 95% CI: 1.20, 1.43). However, in a meta-analysis of follow-up studies by Patel et al [23], the per allele hazard ratio of the 9p21 variants for fatal and non-fatal adjudicated MI was 1.09 (95% CI: 1.03–1.16). The latter is the same as what we report in this study, though the meta-analysis includes earlier reports from some of our studies. One explanation for this inconsistency is the incidence-prevalence bias. Most GWAS for coronary artery disease to date have consisted of cross-sectional case-control studies, a design that over represents patients who survived their MI or CHD event. Using data from five population based cohort studies we found that the reported risk alleles for this locus are associated with longer survival after MI. This finding that was previously reported as well [23–25] supports the conjecture. Thus, the high prevalence of the risk allele in various types of cross-sectional analyses may not be due entirely to a high risk of experiencing MI or CHD, but also to an improved chance of survival after MI.

The molecular biology behind the protective effect of the risk alleles at 9p21 is yet unclear, however, there is a growing body of evidence to show that 9p21 locus is only increasing the risk of CHD for the first event and not for the subsequent events. For instance, Patel et al found no association with subsequent CHD events in a recent meta-analysis of 25,163 individuals with established CHD [23]. Thus, it could be concluded that 9p21 locus is contributing to the formation and progression of plaques and not to their instability prior to events; therefore, the association is merely observed in early stages of the disease. This is in agreement with the report by Palomaki [26] that suggests a diminished effect of 9p21 locus by age, a finding that is confirmed by Patel et al for secondary events. It should be noted that the mean age of participants was more than 70 years old in two and more than 60 years old in four of the participating cohorts. In this context, the older mean age of our population could be another reason why our findings do not replicate known loci such as 9p21.Our study is the largest collection of population-based prospective GWAS on incident MI and CHD and includes high quality genotyping and phenotyping data from well-known cohort studies in the field of cardiovascular disease. Moreover, similar case definitions for MI and CHD, comparable quality control for genotyped data, harmonized imputation strategies and collaboratively designed analysis plans are further strengths of our study. Despite these strengths, there are several limitations that merit discussion. First, nearly all studies who contributed to our GWAS are also members of the CARDIoGRAMPlusC4D Consortium [27], however, they have used only their prevalent cases in CARDIoGRAMPlusC4D project and therefore there is no overlap between the two GWAS. Second, since our sample size was limited, further susceptibility variants of weaker effects may have been missed in our study. Third, we have tried to use consistent definitions for MI, however, slight differences exist between the definitions for CHD. This might have introduced heterogeneity in our case definition. Finally, our findings may not be directly generalizable to non-European populations.

A potential clinical application of risk alleles identified from GWAS is the prospective prediction of cardiovascular disease. To date, the totality of evidence from prospective studies suggests that there is only modest, independent prediction of increased cardiovascular disease risk using genetic information with small to modest incremental reclassification for prediction beyond the known clinical CVD risk scores [28]. This lack of success has been attributed to the small percentage of variance explained by known genetic factors. However, our results also suggest that genetic risk prediction needs to consider differences in genetic variants that predict the risk of cardiovascular disease in prospective and cross-sectional settings.

In summary, using the largest collection of population- based prospective genome-wide association studies we have identified *QKI* as a potential locus for incident myocardial infarction. Furthermore, we have shown that the genes associated with risk of cardiovascular disease may differ in effect size when studied in a cross-sectional case-control versus cohort settings. The role of 9p21 locus may be complex, increasing the risk of incident MI and decreasing mortality among those with CHD. This highlights the importance of examining longitudinal cohort studies in the study of etiology even for genetic factors. These findings may have implications for application of genetic variants in risk estimation for cardiovascular disease, an effort that so far has not provided strong evidence for incremental risk prediction by genetic markers.

## Supporting Information

S1 File Supplementary document: Methods, acknowledgment and funding for the participating studies. (DOCX)

S1 Fig a. QQ Plot for Discovery GWAS MI. b. QQ Plot for Discovery GWAS CHD.
(ZIP)

S2 Fig a. Log-plot for Discovery GWAS MI. b. Log-plot for Discovery GWAS CHD.
(ZIP)

S1 Table Phenotype description of the studies in stage I. (DOCX)

S2 Table Phenotype description of the studies in stage II. (DOCX)

S3 Table Analysis Logistics of the studies in stage I. (DOCX)

S4 Table Analysis Logistics of the studies in stage II. (DOCX)

S5 Table Genotyping/imputation/QC specifics of the studies in stage I. (DOCX)

S6 Table Genotyping/imputation/QC specifics of the studies in stage II. (DOCX)

S7 Table Basic description of the study population for the survival after MI analysis. (DOCX)

S8 Table Basic description of the cohort studies in stage II. (DOCX)

S9 Table Basic description of the case-control studies in stage II. (DOCX)

S10 Table Association of the SNPs taken to stage II with MI. (DOCX)

S11 Table Association of the SNPs taken to stage II with CHD. (DOCX)
