# Supplementary material for: Genome-Wide Association Study for Incident Myocardial Infarction and Coronary Heart Disease in Prospective Cohort Studies: The CHARGE Consortium
Source: PLoS One. 2016 Mar 7;11(3):e0144997. doi: 10.1371/journal.pone.0144997 (PMC4780701; doi:10.1371/journal.pone.0144997)
Supplement: S1 File — (DOCX) [file pone.0144997.s001.docx]

**Supplementary document**

Table of Contents

[Supplementary Methods 2](#_Toc430693360)

[Stage I Studies 2](#_Toc430693361)

[*The Age, Gene/Environment Susceptibility-Reykjavik Study (AGES-Reykjavik)* 2](#_Toc430693362)

[*The Atherosclerosis Risk in Communities Study (ARIC)* 2](#_Toc430693363)

[*The Cardiovascular Health Study (CHS)* 3](#_Toc430693364)

[*The Framingham Heart Study (FHS)* 4](#_Toc430693365)

[*The Rotterdam Study* 5](#_Toc430693366)

[Stage II Studies 6](#_Toc430693367)

[*The Health, Aging, and Body Composition (Health ABC) Study* 6](#_Toc430693368)

[*The Nurses’ Health Study (NHS) and The Health Professionals Follow-Up Study (HPFS)* 7](#_Toc430693369)

[*The MOnica Risk, Genetics, Archiving and Monograph (MORGAM) Study* 8](#_Toc430693370)

[*The PROSPER/PHASE Study* 10](#_Toc430693371)

[*The Study of Health in Pomerania (SHIP)* 11](#_Toc430693372)

[*The Women’s Genome Health Study (WGHS)* 12](#_Toc430693373)

[Supplementary Funding/Acknowledgments 13](#_Toc430693374)

[AGES 13](#_Toc430693375)

[ARIC 13](#_Toc430693376)

[CHS 13](#_Toc430693377)

[FHS 14](#_Toc430693378)

[Rotterdam Study 14](#_Toc430693379)

[HealthABC 15](#_Toc430693380)

[MORGAM 15](#_Toc430693381)

[HPFS 17](#_Toc430693382)

[SHIP 17](#_Toc430693383)

[WGHS 18](#_Toc430693384)

[References 19](#_Toc430693385)

# ****Supplementary Methods****

## ****Stage I Studies****

### ***The Age, Gene/Environment Susceptibility-Reykjavik Study (AGES-Reykjavik)***

The AGES study cohort originally comprised a random sample of 30,795 men and women born in 1907-1935 and living in Reykjavik in 1967. A total of 19,381 people attended, resulting in 71% recruitment rate. The study sample was divided into six groups by birth year and birth date within month. One group was designated for longitudinal follow up and was examined in all stages. One group was designated a control group and was not included in examinations until 1991. Other groups were invited to participate in specific stages of the study. Between 2002 and 2006, the AGES/Reykjavik study re-examined 5764 survivors of the original cohort who had participated before in the Reykjavik Study.[[1](#_ENREF_1),[2](#_ENREF_2)] The AGES/Reykjavik Study GWAS was approved by the National Bioethics Committee (VSN: 00‐063) and the Data Protection Authority.

### ***The Atherosclerosis Risk in Communities Study (ARIC)***

The ARIC study is a population-based prospective cohort study of CVD and its risk factors and includes 15,792 persons aged 45-64 years at baseline (1987-89), randomly chosen from four US communities.[[3](#_ENREF_3)] Cohort members completed four clinic examinations, conducted approximately three years apart between 1987 and 1998. For this study, the analysis was restricted to subjects of European descent who were free of prevalent CHD or stroke at baseline.

Affymetrix 6.0 array genotypes were obtained in 8,861 self-identified whites: 734 individuals were excluded for the following reasons: 1) discordant with previous genotype data, 2) genotypic sex did not match phenotypic sex, 3) suspected first-degree relative of an included individual based on genome-wide genotype data, 4) genetic outlier (as assessed by average Identity by State (IBS) using PLINK and >8 standard deviations along any of first 10 principal components in EIGENSTRAT after 5 iterations. For this analysis, 721 individuals with prevalent CHD or stroke at baseline were excluded. SNPs without chromosomal location, monomorphic SNPs, SNPs whose genotype frequencies between 2 freezes differed by p<10^-6^, SNPs with HWE p<10^-6^ or call rate <90% were excluded from analysis. Imputation of ~2.5 million autosomal SNPs in HapMap with reference to release 22 of the CEU sample was conducted using the algorithm implemented in MACH.

### ***The Cardiovascular Health Study (CHS)***

CHS is a population-based observational study of risk factors for clinical and subclinical cardiovascular diseases.[[4](#_ENREF_4)] The study recruited participants 65 years of age and older from 4 US communities in 2 waves: 1989-1990, and 1992-1993. The original predominantly Caucasian cohort of 5201 persons was recruited in 1989-1990 from a random sample of people on Medicare eligibility lists and an additional 687 African-Americans were enrolled subsequently for a total sample of 5888.

DNA was extracted from blood samples drawn on all participants who consented to genetic testing at their baseline examination in 1989-90. A total of 1908 persons were excluded from the GWAS study sample due to the presence at study baseline of coronary heart disease, congestive heart failure, peripheral vascular disease, valvular heart disease, stroke, transient ischemic attack, or lack of available DNA. In 2007-2008, genotyping was performed at the General Clinical Research Center's Phenotyping/Genotyping Laboratory at Cedars-Sinai using the Illumina 370CNV Duo® BeadChip system on the 3980 CHS participants who were free of CVD at baseline. Because the other cohorts were predominantly white, the African American participants were excluded from this analysis to reduce the possibility of confounding by population structure. Participants were further excluded if they had a call rate<=95%. After applying these exclusions, 3,291 participants with genotype and phenotype were available; they constitute the CHS sample for this study.

From a total of 335,887 genotyped autosomal SNPs the following exclusions were applied to identify a final set of 306,655 SNPs: call rate < 97%, HWE P < 10^-5^, > 2 duplicate errors or Mendelian inconsistencies (for reference CEPH trios), heterozygote frequency = 0, SNP not found in HapMap. Imputation was performed using BIMBAM v0.99 with reference to HapMap CEU using release 22, build 36 using one round of imputations and the default expectation-maximization warm-ups and runs.[[5](#_ENREF_5)]

Mortality, hospitalizations, and cardiovascular events were ascertained at annual examinations and 6-month telephone interviews. Data collection included information from medical records, proxy interviews, and death certificates. Cardiovascular events and causes of death were adjudicated by a committee of physicians who were without knowledge of prior examination findings. [[6](#_ENREF_6)]

### ***The Framingham Heart Study (FHS)***

The FHS started in 1948 with 5,209 randomly ascertained participants from Framingham, Massachusetts, United States, who have undergone biannual examinations to investigate cardiovascular diseases and its risk factors.[[7](#_ENREF_7),[8](#_ENREF_8)] In 1,971, the Offspring cohort[[9](#_ENREF_9),[10](#_ENREF_10)] (comprising 5,124 children of the original cohort, and children’s spouses) and in 2002, the Third Generation (consisting of 4,095 children of the Offspring cohort), were recruited.[[11](#_ENREF_11)] The current study was conducted in 4134 participants of the Original and Offspring cohorts, who were free of CHD at the time of their blood draw for a DNA sample. Of these, 165 developed MI and 201 developed CHD after their DNA draw. An endpoint Committee consisting of three physicians reviewed all suspected cardiovascular disease events to confirm occurrence of the event, using previously agreed criteria. MI in this project was defined as a recognized non-fatal MI diagnosed with documented evidence from EKG or elevated enzymes; CHD was defined as recognized fatal or non-fatal MI, coronary insufficiency or the first fatal CHD event.

Genotyping was conducted for the SNP Health Association Resource (SHARe) project (<http://www.ncbi.nlm.nih.gov/projects/gap/cgi-bin/study.cgi?study_id=phs000007.v10.p5>) using the Affymetrix 500K mapping array (250K Nsp and 250K Sty arrays) and the Affymetrix 50K supplemental gene focused array on a total of 9,274 individuals from all three cohorts. Genotyping resulted in 503,551 SNPs with successful call rate >95% and HWE P>1.0×10^-6^ in 8,481 individuals with call rate >97%. Imputation of 2,543,887 autosomal SNPs in HapMap release 22, build 36, CEU sample was conducted using the algorithm implemented in MACH (version 1.0.15). From a total of 534,982 genotyped autosomal SNPs in Framingham, 378,163 SNPs were used in imputation after filtering out 15,586 SNPs (HWE P<1.0×10^-6^), 64,511 SNPs (missingness >0.03), 45,361 SNPs (mishap P<1.0×10^-9^), 4,857 SNPs (>100 Mendelian errors), 67,269 SNPs (frequency <0.01), 2 SNPs (due to strand issues upon merging data with HapMap), and a further 13,394 SNPs that were not present on HapMap. We used 200 biologically unrelated participants to estimate the parameters of the imputation model and subsequently applied the estimated parameters to obtain imputed SNPs for all 8,481 participants. To evaluate population substratification, we conducted principal component analyses using EIGENSTRAT [[12](#_ENREF_12)] on the genotypes from 882 unrelated participants. We estimated the first 10 principal components and applied the loadings of these components to all genotyped participants. Finally, we evaluated whether any of these principal components were associated with MI or CHD. Principal components 3 and 4 were found to be associated with both MI and CHD. All analyses of individual SNPs adjusted for sex, age, generation (Original or Offspring Cohort), and principal components 3 and 4 using Cox proportional hazards models with robust standard errors calculated by clustering on families to account for familial correlations. The Framingham Heart Study was approved by the institutional review boards of Boston University and the National Institutes of Health. All participants provided written informed consent.

### ***The Rotterdam Study***

The Rotterdam Study (RS) is a prospective, population-based cohort study on determinants of several chronic diseases in older adults.[[13](#_ENREF_13),[14](#_ENREF_14)] In brief, 10275 inhabitants of Ommoord, a district of Rotterdam in the Netherlands, who were 55 years or over, were invited to participate in this study and 7983 agreed to participate (78%). Written informed consent was obtained and the Medical Ethics Committee of Erasmus Medical Center approved the study. The baseline examinations took place from 1990-1993. Participants were visited at home for an interview. Information on current health status, medical history, and use of medication were obtained during the interview. The interview was followed by two visits at the research center for blood sampling and further examinations.

The version 3 Illumina Infinium II HumanHap550 SNP chip array was used to conduct genotyping among self-reported Caucasians. Genotyping was successful in 6,240 individuals with a sample call rate >97.5%. SNPs with a call rate <95% and HWE p<10^-6^ were excluded. The final dataset for this analysis comprised data on 530,683 SNPs in 5,974 RS participants. Imputation was conducted using the algorithm implemented in MACH. To obtain imputed data, more restrictive SNP filters including a minor allele frequency >0.01, SNP call rate >0.98, and HWE p-value >1×10^-6^ were applied and 491,875 passed the filters. In total 2,586,725 SNPs were imputed using phased haplotypes of HapMap CEU trios.

To identify incident myocardial infarction and coronary heart disease, we collected information from baseline (1990 - 1993) until January 1, 2005. Fatal or non-fatal MI reported by general practitioners in the research area, letters from medical specialists and discharge reports for hospitalized patients were the sources of information used. Two research physicians coded the events independently and in case of disagreement the consensus was made in a separate session. Finally a specialist whose judgment was considered final verified the coding. [[15](#_ENREF_15),[16](#_ENREF_16)]

## ****Stage II Studies****

### ***The Health, Aging, and Body Composition (Health ABC) Study***

The Health, Aging, and Body Composition (Health ABC) Study is a prospective cohort study investigating the associations between body composition, weight-related health conditions, and incident functional limitation in older adults. Health ABC enrolled well-functioning, community-dwelling black (n=1281) and white (n=1794) men and women aged 70-79 years between April 1997 and June 1998. Participants were recruited from a random sample of all white and black Medicare eligible residents in the Pittsburgh, PA, and Memphis, TN, metropolitan areas. Of 3,075 participants at baseline, 1661 Caucasians had both genotype and phenotype data available for analysis.

Genomic DNA was extracted from buffy coat collected using PUREGENE® DNA Purification Kit during the baseline exam. Genotyping was performed by the Center for Inherited Disease Research (CIDR) using the Illumina Human1M-Duo BeadChip system. Samples were excluded from the dataset for the reasons of sample failure, genotypic sex mismatch, and first-degree relative of an included individual based on genotype data. Genotyping was successful for 1,151,215 SNPs in 1663 unrelated Caucasian individuals. Imputation was done for the autosomes using the MACH software version 1.0.16. SNPs with minor allele frequency ≥ 1%, call rate ≥97% and HWE p≥10-6 were used for imputation. HapMap II phased haplotypes were used as reference panels. For EAs, genotypes were available on 914,263 high quality SNPs for imputation based on the HapMap CEPH reference panel (release 22, build 36). A total of 2,543,887 are available for analysis.

### ***The Nurses’ Health Study (NHS) and The Health Professionals Follow-Up Study (HPFS)***

The Nurses’ Health Study (NHS) cohort was established in 1976 when 121,700 female registered nurses aged 30 to 55 years residing in 11 U.S. states completed a mailed questionnaire on their medical history and lifestyle characteristics.[[17](#_ENREF_17)] The women have since received follow-up questionnaires biennially to update information on exposures and newly diagnosed illnesses. Among the 32,826 participants who provided blood samples and who were without cardiovascular disease or cancer at blood draw, incident cases of non-fatal myocardial infarction (MI) and fatal CHD occurring after blood draw until 2004 were selected and matched with 2 randomly selected controls using risk-set sampling, matching factors were age and smoking. [[18](#_ENREF_18)]

The Health Professionals Follow-Up Study (HPFS) was initiated in 1986 when 51,529 male health professionals between 40 and 75 years of age completed a FFQ and a medical history questionnaire. The participants have been followed with repeated questionnaires on lifestyle and health every 2 years. Among the 18,225 participants who provided blood samples and who were without cardiovascular disease or cancer at blood draw, incident cases of non-fatal myocardial infarction (MI) and fatal CHD occurring after blood draw until 2004 were selected and matched with 2 randomly selected controls using risk-set sampling, matching factors were age and smoking. [[18](#_ENREF_18)]

The participants of both studies have been followed with repeated questionnaires on lifestyle and health every 2 years. Participants who had reported an incident CHD on the follow-up questionnaire were contacted for confirmation and permission to review medical records was requested. Medical records for deceased participants were also sought for deaths that were identified by families and postal officials and through the National Death Index. Physicians blinded to the participant’s questionnaire reports reviewed all medical records. Cases of MI and fatal CHD were identified primarily through review of medical records, as previously described.[[17](#_ENREF_17),[19](#_ENREF_19)]

For both studies, DNA was extracted for genotyping in 2008 at the Merck Research Laboratories, North Wales, PA, using the Affymetrix Genome-Wide Human 6.0 array. QC criteria used to define unsuccessful genotyping were a call rate < 97%, sex mismatch, and HWE p value<1.0×10^-4^ in controls. Analyses based on principal components were conducted to assess self-reported race. Subsequent analysis sample was restricted to subjects of European ancestry. Self-reported "white" samples with substantial similarity to non-European reference samples (either the HapMap YRI or CHB+JPT samples) were excluded (n=16). Three eigenvectors were included as covariates to adjust for potential population stratification in the final sample. Imputations were done using MACH software based on the 12K SNPs reported previously. [[20](#_ENREF_20),[21](#_ENREF_21)]

### ***The MOnica Risk, Genetics, Archiving and Monograph (MORGAM) Study***

The MOnica Risk, Genetics, Archiving and Monograph (MORGAM) Study ([www.thl.fi/morgam](http://www.thl.fi/morgam)) is a collaboration of prospective follow-up studies of the respondents of representative population samples in different European countries for CHD and stroke.[[22](#_ENREF_22)] The cohort descriptions, including the follow-up procedures for each cohort, have been published elsewhere.[[23](#_ENREF_23)] This study includes cohorts from Finland (ATBC (FIN-ATB), FINRISK92 and FINRISK97 (FIN-EAS/WES)), France (PRIME (FRA-LIL, FRA-STR, FRA-TOU)), Northern Sweden (SWE-NSW cohorts 02 and 03) and UK (PRIME/Belfast (UNK-BEL)). The total size of these predominantly Caucasian cohorts is 33 281 men and women. The baseline examinations, including blood sampling for DNA, were carried out between years 1990 and 1997. The average follow-up period of the cohorts varied between 6 and 15 years.

For its genetic component, MORGAM has a case-cohort design.[[23](#_ENREF_23)] Excluded from this study are those who had a documented or self-reported MI at baseline or (b) did not have DNA available for a successful genotyping. Genotyping methods for this study: Genotyping was carried out at the National Institute for Health and Welfare, Helsinki, Finland. Several sample- and plate-specific quality control measures were implemented to minimize errors with sample and plate handling. The sample-specific measures included gender-specific PCR test, success of microsatellite 5-plex genotyping, and DNA quantity and quality testing with picogreen. The plate-specific measures included 2% water control samples, 2% known duplicate samples, and 2% CEPH control samples in unique positions in each 96-well plate. In addition, genotyping quality was assessed from 5% blind duplicate samples in each 96-well plate.[[24](#_ENREF_24)] For 234 samples with low DNA yield, DNA was amplified prior to genotyping as previously described. [[24](#_ENREF_24),[25](#_ENREF_25)]

A total of 52 SNPs were selected for the study. These included 2-3 SNPs from each of 15 loci associated with MI, and 6 loci associated with CHD. We successfully genotyped 49 of the SNPs, using Sequenom MassARRAY System and iPLEX Gold chemistry (Sequenom, San Diego, California) with standard protocol. Genotype clusters were manually reviewed using the Typer 4.0 software (Sequenom), and genotype calls were corrected when necessary. For each of 11 cohort-geographic region combinations we tested that the genotype distribution in the subcohort was in Hardy-Weinberg equilibrium (HWE). The HWE p-value for four SNPs was <0.01 for 2-3/11 cohort-geographic region combinations: rs1489719, rs16841920, rs17777478, and rs6504582. All markers had genotyping success rate >95%, with an average genotyping success rate of 97.6%. Among a total of 15,635 successful blind duplicate genotype pairs, one discrepancy was identified (concordance of 99.99%).

The follow-up and diagnostic procedures vary between the cohorts, and have been described previously.[[23](#_ENREF_23)] As a rough summary, in Finland and Sweden, the follow-up was based on record linkage with national computerized causes-of-death register, hospital discharge register and a regional coronary event register. In France and UK the surviving cohort members were contacted annually. The end-point diagnoses were scrutinized by the study teams using all available medical information. For a part of the cases in Finland and Sweden, the official cause of death or the hospital diagnosis was used, after a validation study of the quality of such diagnoses.

### ***The PROSPER/PHASE Study***

All data come from the PROspective Study of Pravastatin in the Elderly at Risk (PROSPER). A detailed description of the study has been published elsewhere.[[26](#_ENREF_26),[27](#_ENREF_27)] PROSPER was a prospective multicenter randomized placebo-controlled trial to assess whether treatment with pravastatin diminishes the risk of major vascular events in elderly. Between December 1997 and May 1999, we screened and enrolled subjects in Scotland (Glasgow), Ireland (Cork), and the Netherlands (Leiden). Men and women aged 70-82 years were recruited if they had pre-existing vascular disease or increased risk of such disease because of smoking, hypertension, or diabetes. A total number of 5,804 subjects were randomly assigned to pravastatin or placebo. A large number of prospective tests were performed including Biobank tests and cognitive function measurements. A whole genome wide screening has been performed in the sequential PHASE project with the use of the Illumina 660K beadchip. Of 5,763 subjects DNA was available for genotyping. Genotyping was performed with the Illumina 660K beadchip, after QC (call rate <95%) 5,244 subjects and 557,192 SNPs were left for analysis. These SNPs were imputed to 2.5 million SNPs based on the HAPMAP built 36 with MACH imputation software.

### ***The Study of Health in Pomerania (SHIP)***

The Study of Health in Pomerania (SHIP) is a cohort study in West Pomerania, the north-east area of Germany.[[28](#_ENREF_28),[29](#_ENREF_29)] A sample from the population aged 20 to 79 years was drawn from population registries. First, the three cities of the region (with 17,076 to 65,977 inhabitants) and the 12 towns (with 1,516 to 3,044 inhabitants) were selected, and then 17 out of 97 smaller towns (with less than 1,500 inhabitants), were drawn at random. Second, from each of the selected communities, subjects were drawn at random, proportional to the population size of each community and stratified by age and gender. Only individuals with German citizenship and main residency in the study area were included. Finally, 7,008 subjects were sampled, with 292 persons of each gender in each of the twelve five-year age strata. In order to minimize drop-outs by migration or death, subjects were selected in two waves. The net sample (without migrated or deceased persons) comprised 6,267 eligible subjects. Selected persons received a maximum of three written invitations. In case of non-response, letters were followed by a phone call or by home visits if contact by phone was not possible. The SHIP population finally comprised 4,308 participants (corresponding to a final response of 68.7%). From these 4,308 individuals, we had 4,081 with GWAS information. From them, we excluded 194 individuals with prevalent myocardial infarction (n=152) and/or heart surgery (n=76) and 4 individuals with no information regarding date of the event. The final number for the analyses was 3,883 individuals. In 133 subjects an incident fatal or non-fatal MI was diagnosed after the baseline examination (1997 - 2001) and until 04.08.12 (the last day of information collected). Fatal or non-fatal MI was defined by self-report during follow-up interviews of the participants, reports by participants’ general practitioners, death certificate and information about hospitalized patients in the hospital’s university.

The SHIP samples were genotyped using the Affymetrix Genome-Wide Human SNP Array 6.0. Hybridisation of genomic DNA was done in accordance with the manufacturer’s standard recommendations. The genetic data analysis workflow was created using the Software InforSense. Genetic data were stored using the database Caché (InterSystems). Genotypes were determined using the Birdseed2 clustering algorithm. For quality control purposes, several control samples where added. On the chip level, only subjects with a genotyping rate on QC probesets (QC callrate) of at least 86% were included. Finally, all arrays had a sample callrate > 92%. The overall genotyping efficiency of the GWA was 98.55 %. Imputation of genotypes in SHIP was performed with the software IMPUTE v0.5.0 based on HapMap II.

### ***The Women’s Genome Health Study (WGHS)***

The Women’s Genome Health Study (WGHS) is a prospective cohort of female North American health care professionals representing participants in the Women’s Health Study (WHS) trial who provided a blood sample at baseline and consent for blood-based analyses. [[30](#_ENREF_30)] Participants in the WHS were 45 or older at enrollment and free of cardiovascular disease, cancer or other major chronic illness. For the primary WHS endpoints of cardiovascular disease, full medical records were obtained for reported endpoints and reviewed by an endpoints committee of physicians unaware of randomized treatment assignment. The current data are derived from 23,294 WGHS participants for whom whole genome genotype information was available at the time of analysis and self-reported European ancestry could be confirmed by multidimensional scaling analysis of 1443 ancestry informative markers in PLINK v. 1.06. Whole genome genetic analysis was performed with ProbABEL (v. 0.0-6, http://mga.bionet.nsc.ru/ yurii/ABEL/). Additional analysis was performed in R.

# ****Supplementary Funding/Acknowledgments****

### AGES

The Age, Gene/Environment Susceptibility Reykjavik Study has been funded by NIH contract N01-AG-12100, the NIA Intramural Research Program, Hjartavernd (the Icelandic Heart Association), and the Althingi (the Icelandic Parliament).  The study is approved by the Icelandic National Bioethics Committee, (VSN: 00-063) and the Data Protection Authority.  The researchers are indebted to the participants for their willingness to participate in the study.

### ARIC

The Atherosclerosis Risk in Communities Study is carried out as a collaborative study supported by National Heart, Lung, and Blood Institute contracts (HHSN268201100005C, HHSN268201100006C, HHSN268201100007C, HHSN268201100008C, HHSN268201100009C, HHSN268201100010C, HHSN268201100011C, and HHSN268201100012C), R01HL087641, R01HL59367 and R01HL086694; National Human Genome Research Institute contract U01HG004402; and National Institutes of Health contract HHSN268200625226C. The authors thank the staff and participants of the ARIC study for their important contributions. Infrastructure was partly supported by Grant Number UL1RR025005, a component of the National Institutes of Health and NIH Roadmap for Medical Research.

### CHS

This CHS research was supported by NHLBI contracts HHSN268201200036C, HHSN268200800007C, N01HC55222, N01HC85079, N01HC85080, N01HC85081, N01HC85082, N01HC85083, N01HC85086; and NHLBI grants HL080295, HL087652, HL103612, HL105756, HL120393 with additional contribution from the National Institute of Neurological Disorders and Stroke (NINDS). Additional support was provided through AG023629 from the National Institute on Aging (NIA). A full list of principal CHS investigators and institutions can be found at CHS-NHLBI.org/.The provision of genotyping data was supported in part by the National Center for Advancing Translational Sciences, CTSI grant UL1TR000124, and the National Institute of Diabetes and Digestive and Kidney Disease Diabetes Research Center (DRC) grant DK063491 to the Southern California Diabetes Endocrinology Research Center.The content is solely the responsibility of the authors and does not necessarily represent the official views of the National Institutes of Health. The infrastructure for the CHARGE Consortium is supported in part by NHLBI grant HL105756.

### FHS

This research was conducted in part using data and resources from the Framingham Heart Study of the National Heart Lung and Blood Institute of the National Institutes of Health and Boston University School of Medicine. The analyses reflect intellectual input and resource development from the Framingham Heart Study investigators participating in the SNP Health Association Resource (SHARe) project. This work was partially supported by the National Heart, Lung and Blood Institute's Framingham Heart Study (Contract No. N01-HC-25195) and its contract with Affymetrix, Inc for genotyping services (Contract No. N02-HL-6-4278). A portion of this research utilized the Linux Cluster for Genetic Analysis (LinGA-II) funded by the Robert Dawson Evans Endowment of the Department of Medicine at Boston University School of Medicine and Boston Medical Center.

### Rotterdam Study

The Rotterdam Study is supported by the Erasmus Medical Center and Erasmus University Rotterdam; the Netherlands Organization for Scientific Research (NWO); the Netherlands Organization for Health Research and Development (ZonMw); the Research Institute for Diseases in the Elderly (RIDE); the Netherlands Heart Foundation; the Ministry of Education, Culture and Science; the Ministry of Health Welfare and Sports; the European Commission; and the Municipality of Rotterdam. Support for genotyping was provided by the Netherlands Organisation of Scientific Research NWO Investments (nr. 175.010.2005.011, 911-03-012), the Research Institute for Diseases in the Elderly (014-93-015; RIDE2), the Netherlands Genomics Initiative (NGI)/Netherlands Consortium for Healthy Aging (NCHA) project nr. 050-060-810. Abbas Dehghan is supported by NWO grant (veni, 916.12.154) and the EUR Fellowship.

### HealthABC

The Health Aging and Body Composition Study (Health ABC) was funded by the National Institutes of Aging. This research was supported by NIA contracts N01AG62101, N01AG62103, and N01AG62106. The genome-wide association study was funded by NIA grant 1R01AG032098-01A1 to Wake Forest University Health Sciences and genotyping services were provided by the Center for Inherited Disease Research (CIDR). CIDR is fully funded through a federal contract from the National Institutes of Health to The Johns Hopkins University, contract number HHSN268200782096C.

### MORGAM

The MORGAM research was part funded through the European Community's Seventh Framework Programme (FP7/2007-2013), ENGAGE project, grant agreement HEALTH-F4-2007-201413. KS has been supported by the Academy of Finland Centre of Excellence in Complex Disease Genetics. We warmly thank all the participants in the MORGAM study. Sites and key personnel of contributing MORGAM Centres:

Finland: FINRISK, National Public Health Institute, Helsinki: V. Salomaa (principal investigator), A. Juolevi, E. Vartiainen, P. Jousilahti; ATBC, National Public Health Institute, Helsinki: J. Virtamo (principal investigator), H. Kilpeläinen; MORGAM Data Centre, National Public Health Institute, Helsinki: K. Kuulasmaa (head), Z. Cepaitis, A. Haukijärvi, B. Joseph, J. Karvanen, S. Kulathinal, M. Niemelä, O. Saarela; MORGAM Central Laboratory, National Public Health Institute, Helsinki: M. Perola (responsible person), L. Peltonen (former responsible person), K. Silander, P. Wagner, A. Wikman,M. Jussila, O Törnwall, M. Alanne, P. Laiho;

France : National Coordinating Centre, National Institute of Health and Medical Research (U258), Paris: P. Ducimetière (national coordinator), A. Bingham; PRIME/Strasbourg, Department of Epidemiology and Public Health, EA 3430, University of Strasbourg, Faculty of Medicine, Strasbourg: D. Arveiler (principal investigator), B. Haas, A. Wagner; PRIME/Toulouse, Department of Epidemiology, Faculty of Medicine, Toulouse-Purpan, Toulouse: J. Ferrières (Principal Investigator), J-B. Ruidavets, V. Bongard, D. Deckers, M. Massabuau, S. Barrere, M. Souviraa;
PRIME/Lille, Department of Epidemiology and Public Health, Pasteur Institute of Lille: P. Amouyel (principal investigator), M. Montaye, B. Lemaire, S. Beauchant, D. Cottel, C. Graux, N. Marecaux, C. Steclebout, S. Szeremeta; MORGAM Laboratory, INSERM U525, Paris: F. Cambien (responsible person), L. Tiret, V. Nicaud, D. Tregouet;

Sweden: Northern Sweden, Umeå University Hospital, Department of Medicine, Umeå: S. Söderberg (principal investigator), P-G. Wiklund (former principal investigator), B. Stegmayr (principal investigator), K. Asplund (former principal investigator), S. Nasic, G. Rönnberg, Å. Johansson, V. Lundberg, E. Jägare-Westerberg, T. Messner;

United Kingdom: PRIME/Belfast, Queen's University Belfast, Belfast, Northern Ireland: F Kee (principal investigator), A. Evans (former principal investigator) J. Yarnell, E. Gardner;
MORGAM Coordinating Centre, Queen's University Belfast, Belfast, Northern Ireland: A. Evans (MORGAM coordinator), S. Cashman;

MORGAM Management Group: K. Kuulasmaa (chair), A. Evans (chair), S. Blankenberg (Hamburg, Germany), M. Ferrario (Varese, Italy) , F. Kee, A. Palotie (Hinxton, England), M. Perola, A. Peters (Munich, Germany), V. Salomaa, D. Tregouet, H. Tunstall-Pedoe (Dundee, Scotland); Previous members K. Asplund (Stockholm Sweden), F. Cambien, L. Peltonen, D. Shields (Dublin, Ireland), B. Stegmayr, P.-G. Wiklund (Sweden).

### HPFS

The HPFS CHD case-control study was supported by HL35464, and CA55075 from the National Institutes of Health, Bethesda, MD, with additional support for genotyping from Merck/Rosetta Research Laboratories, North Wales, PA.

NHS

The NHS CHD case-control study was supported by CA87969 and HL34594 from the National Institutes of Health, Bethesda, MD, with additional support for genotyping from Merck/Rosetta Research Laboratories, North Wales, PA.

### SHIP

SHIP is part of the Community Medicine Research net of the University of Greifswald, Germany, which is funded by the Federal Ministry of Education and Research (grants no. 01ZZ9603, 01ZZ0103, and 01ZZ0403), the Ministry of Cultural Affairs as well as the Social Ministry of the Federal State of Mecklenburg-West Pomerania, and the network ‘Greifswald Approach to Individualized Medicine (GANI_MED)’ funded by the Federal Ministry of Education and Research (grant 03IS2061A). Genome-wide data have been supported by the Federal Ministry of Education and Research (grant no. 03ZIK012) and a joint grant from Siemens Healthcare, Erlangen, Germany and the Federal State of Mecklenburg- West Pomerania. The University of Greifswald is a member of the ‘Center of Knowledge Interchange’ program of the Siemens AG and the Caché Campus program of the InterSystems GmbH.

### WGHS

The WGHS is supported by HL 043851 and HL080467 from the National Heart, Lung, and Blood Institute and CA 047988 from the National Cancer Institute, the Donald W. Reynolds Foundation and the Fondation Leducq, with collaborative scientific support and funding for genotyping provided by Amgen. Additional funding for collecting and validating incident myocardial infarction and coronary heart disease was provided by HL099355 under the American Recovery and Reinvestment Act (ARRA).

# ****References****

1. Harris TB, Launer LJ, Eiriksdottir G, Kjartansson O, Jonsson PV, et al. (2007) Age, Gene/Environment Susceptibility-Reykjavik Study: multidisciplinary applied phenomics. Am J Epidemiol 165: 1076-1087.

2. Sigurdsson E, Thorgeirsson G, Sigvaldason H, Sigfusson N (1995) Unrecognized myocardial infarction: epidemiology, clinical characteristics, and the prognostic role of angina pectoris. The Reykjavik Study. Ann Intern Med 122: 96-102.

3. (1989) The Atherosclerosis Risk in Communities (ARIC) Study: design and objectives. The ARIC investigators. Am J Epidemiol 129: 687-702.

4. Fried LP, Borhani NO, Enright P, Furberg CD, Gardin JM, et al. (1991) The Cardiovascular Health Study: design and rationale. Ann Epidemiol 1: 263-276.

5. Servin B, Stephens M (2007) Imputation-based analysis of association studies: candidate regions and quantitative traits. PLoS Genet 3: e114.

6. (1995) Design, rational, and baseline characteristics of the Prospective Pravastatin Pooling (PPP) project--a combined analysis of three large-scale randomized trials: Long-term Intervention with Pravastatin in Ischemic Disease (LIPID), Cholesterol and Recurrent Events (CARE), and West of Scotland Coronary Prevention Study (WOSCOPS). Am J Cardiol 76: 899-905.

7. Dawber TR, Kannel WB, Lyell LP (1963) An approach to longitudinal studies in a community: the Framingham Study. Ann N Y Acad Sci 107: 539-556.

8. Dawber TR, Meadors GF, Moore FE, Jr. (1951) Epidemiological approaches to heart disease: the Framingham Study. Am J Public Health Nations Health 41: 279-281.

9. Feinleib M, Kannel WB, Garrison RJ, McNamara PM, Castelli WP (1975) The Framingham Offspring Study. Design and preliminary data. Prev Med 4: 518-525.

10. Kannel WB, Feinleib M, McNamara PM, Garrison RJ, Castelli WP (1979) An investigation of coronary heart disease in families. The Framingham offspring study. Am J Epidemiol 110: 281-290.

11. Splansky GL, Corey D, Yang Q, Atwood LD, Cupples LA, et al. (2007) The Third Generation Cohort of the National Heart, Lung, and Blood Institute's Framingham Heart Study: design, recruitment, and initial examination. Am J Epidemiol 165: 1328-1335.

12. Price AL, Patterson NJ, Plenge RM, Weinblatt ME, Shadick NA, et al. (2006) Principal components analysis corrects for stratification in genome-wide association studies. Nat Genet 38: 904-909.

13. Hofman A, Grobbee DE, de Jong PT, van den Ouweland FA (1991) Determinants of disease and disability in the elderly: the Rotterdam Elderly Study. Eur J Epidemiol 7: 403-422.

14. Hofman A, van Duijn CM, Franco OH, Ikram MA, Janssen HL, et al. (2011) The Rotterdam Study: 2012 objectives and design update. Eur J Epidemiol 26: 657-686.

15. de Bruyne MC, Mosterd A, Hoes AW, Kors JA, Kruijssen DA, et al. (1997) Prevalence, determinants, and misclassification of myocardial infarction in the elderly. Epidemiology 8: 495-500.

16. van der Meer IM, Bots ML, Hofman A, del Sol AI, van der Kuip DA, et al. (2004) Predictive value of noninvasive measures of atherosclerosis for incident myocardial infarction: the Rotterdam Study. Circulation 109: 1089-1094.

17. Colditz GA, Manson JE, Hankinson SE (1997) The Nurses' Health Study: 20-year contribution to the understanding of health among women. J Womens Health 6: 49-62.

18. Prentice RL, Breslow NE (1978) Retrospective studies and failure time models. Biometrika 65: 153-158.

19. Rimm EB, Giovannucci EL, Willett WC, Colditz GA, Ascherio A, et al. (1991) Prospective study of alcohol consumption and risk of coronary disease in men. Lancet 338: 464-468.

20. Yu K, Wang Z, Li Q, Wacholder S, Hunter DJ, et al. (2008) Population substructure and control selection in genome-wide association studies. PLoS One 3: e2551.

21. Jensen MK, Pers TH, Dworzynski P, Girman CJ, Brunak S, et al. (2011) Protein interaction-based genome-wide analysis of incident coronary heart disease. Circ Cardiovasc Genet 4: 549-556.

22. Evans A, Salomaa V, Kulathinal S, Asplund K, Cambien F, et al. (2005) MORGAM (an international pooling of cardiovascular cohorts). Int J Epidemiol 34: 21-27.

23. Kulathinal S, Karvanen J, Saarela O, Kuulasmaa K (2007) Case-cohort design in practice - experiences from the MORGAM Project. Epidemiol Perspect Innov 4: 15.

24. Silander K, Alanne M, Kristiansson K, Saarela O, Ripatti S, et al. (2008) Gender differences in genetic risk profiles for cardiovascular disease. PLoS One 3: e3615.

25. Silander K, Komulainen K, Ellonen P, Jussila M, Alanne M, et al. (2005) Evaluating whole genome amplification via multiply-primed rolling circle amplification for SNP genotyping of samples with low DNA yield. Twin Res Hum Genet 8: 368-375.

26. Shepherd J, Blauw GJ, Murphy MB, Bollen EL, Buckley BM, et al. (2002) Pravastatin in elderly individuals at risk of vascular disease (PROSPER): a randomised controlled trial. Lancet 360: 1623-1630.

27. Shepherd J, Blauw GJ, Murphy MB, Cobbe SM, Bollen EL, et al. (1999) The design of a prospective study of Pravastatin in the Elderly at Risk (PROSPER). PROSPER Study Group. PROspective Study of Pravastatin in the Elderly at Risk. Am J Cardiol 84: 1192-1197.

28. John U, Greiner B, Hensel E, Ludemann J, Piek M, et al. (2001) Study of Health In Pomerania (SHIP): a health examination survey in an east German region: objectives and design. Soz Praventivmed 46: 186-194.

29. Volzke H, Alte D, Schmidt CO, Radke D, Lorbeer R, et al. (2011) Cohort profile: the study of health in Pomerania. Int J Epidemiol 40: 294-307.

30. Ridker PM, Chasman DI, Zee RY, Parker A, Rose L, et al. (2008) Rationale, design, and methodology of the Women's Genome Health Study: a genome-wide association study of more than 25,000 initially healthy american women. Clin Chem 54: 249-255.
