# Supplementary material for: Genome-Wide Association Study for Incident Myocardial Infarction and Coronary Heart Disease in Prospective Cohort Studies: The CHARGE Consortium
Source: PLoS One. 2016 Mar 7;11(3):e0144997. doi: 10.1371/journal.pone.0144997 (PMC4780701; doi:10.1371/journal.pone.0144997)
Supplement: S1 Table — (DOCX) [file pone.0144997.s004.docx]

### ****S1 Table - Phenotype description of the studies in stage I****

|  | ***AGES*** | ***ARIC*** | ***CHS*** | ***FHS*** | ***The Rotterdam Study*** |
| --- | --- | --- | --- | --- | --- |
| **Participants with phenotype & genotype** | 3219 | 7406 | 3291 | 4134 | 5974 |
| **MI definition** | Fatal or non-fatal MI | Fatal or non-fatal MI | Fatal or non-fatal MI | Fatal or non-fatal MI | Fatal or non-fatal MI |
| **CHD definition** | Fatal or non-fatal MI; CABG: PTCA | Fatal or non-fatal MI, fatal CHD, silent MI | Fatal or non-fatal MI, fatal CHD, sudden death | Fatal or non-fatal MI, fatal CHD, sudden death, coronary insufficiency | Fatal or non-fatal MI, fatal CHD, sudden death |
| **Surveillance Method** | Hospital Records | Regular examinations and telephone interviews, plus hospital records, and death certificates | Regular examinations and telephone interviews, plus medical records, Medicare data, proxy interviews, and death certificates. | Regular examinations, followed up with hospital records | . Hospital records, death certificates, regular examinations |
