# Supplementary material for: Genome-Wide Association Study for Incident Myocardial Infarction and Coronary Heart Disease in Prospective Cohort Studies: The CHARGE Consortium
Source: PLoS One. 2016 Mar 7;11(3):e0144997. doi: 10.1371/journal.pone.0144997 (PMC4780701; doi:10.1371/journal.pone.0144997)
Supplement: S2 Table — (DOCX) [file pone.0144997.s005.docx]

### ****S2 Table - Phenotype description of the studies in stage II****

|  | ***Health ABC*** | ***HPFS*** | ***MORGAM*** | ***NHS*** | ***PROSPER*** | ***SHIP*** | ***WGHS*** |
| --- | --- | --- | --- | --- | --- | --- | --- |
| **Participants with phenotype & genotype** | 1661 | 1313 | 3343 * | 1409 | 5243 | 3883 | 23294 |
| **MI definition** | Fatal or non-fatal MI | NA | Definite or possible AMI or coronary death, unstable angina pectoris | NA | Fatal or non-fatal MI | Fatal or non-fatal MI | Fatal or non-fatal MI |
| **CHD definition** | MI, hospitalized angina, CHD death | Fatal or non-fatal MI, fatal CHD or sudden death | definite or possible AMI or coronary death, unstable angina pectoris, unclassifiable death, cardiac revascularization | Fatal or non-fatal MI, fatal CHD or sudden death | Fatal or non-fatal MI | NA | Fatal or non-fatal MI, or CHD death |
| **Surveillance Method** | Review of medical records by endpoint committee (all MDs) | Review of medical records and death certificates by endpoint committee | See description of MORGAM in the text | Review of medical records and death certificates by endpoint committee | Review of medical records by endpoint committee (all MDs) | NA | Review of medical records by endpoint committee (all MDs) |

*Successful genotyping for 95% of the SNPs
