# Supplementary material for: Genome-Wide Association Study for Incident Myocardial Infarction and Coronary Heart Disease in Prospective Cohort Studies: The CHARGE Consortium
Source: PLoS One. 2016 Mar 7;11(3):e0144997. doi: 10.1371/journal.pone.0144997 (PMC4780701; doi:10.1371/journal.pone.0144997)
Supplement: S3 Table — (DOCX) [file pone.0144997.s006.docx]

### ****S3 Table - Analysis Logistics of the studies in stage I****

|  | ***AGES*** | ***ARIC*** | ***CHS*** | ***FHS*** | ***RS*** |
| --- | --- | --- | --- | --- | --- |
| **Adjustments** | Age and sex | Age and sex | Age, sex and clinic: as adjustment in genotype-phenotype model | Age, sex, cohort (1st or 2nd generation), principal components 3 and 4 | Age and sex |
| **Analysis method** | Cox regression, excluding individuals with prevalent events | Cox regression; Entry at baseline. Censoring at death or loss to follow-up. Covariates from baseline. | Cox regression; Entry at time of blood draw used for genotyping (baseline). Covariates come from baseline exam. Censoring at death or loss to follow-up | Cox regression with clustering on families and robust standard errors; entry for follow up at time of blood draw for DNA; censoring at death or loss to follow up. Covariates from exam of DNA draw | Cox regression;  Entry at time of blood draw used for genotyping (baseline). Covariates come from baseline exam. Censoring at death or loss to follow-up |
| **Analysis software** | R version 2.7 (http://www.r-project.org) | ProbABEL(http://mga.bionet.nsc.ru/~yurii/ABEL/) | R version 2.7 ([www.r-project.org](http://www.r-project.org)) | coxph function in R ([www.r-project.org](http://www.r-project.org/)) | ProbABEL(http://mga.bionet.nsc.ru/~yurii/ABEL/) |
