# Supplementary material for: Genome-Wide Association Study for Incident Myocardial Infarction and Coronary Heart Disease in Prospective Cohort Studies: The CHARGE Consortium
Source: PLoS One. 2016 Mar 7;11(3):e0144997. doi: 10.1371/journal.pone.0144997 (PMC4780701; doi:10.1371/journal.pone.0144997)
Supplement: S4 Table — (DOCX) [file pone.0144997.s007.docx]

### ****S4 Table - Analysis Logistics of the studies in stage II****

|  | ***Health ABC*** | ***HPFS*** | ***MORGAM*** | ***NHS*** | ***PROSPER*** | ***SHIP*** | ***WGHS*** |
| --- | --- | --- | --- | --- | --- | --- | --- |
| **Adjustments** | Age, sex, clinic, PC1, chd prevalence | Nested case-control study, matched on age and smoking. Additional adjustment for top 3 eigenvectors | Age at baseline, sex, cohort | (Nested case-control study, matched on age and smoking). Additional adjustment for top 3 eigenvectors | Age, sex, statin use, and PCI | Age, sex | Age |
| **Analysis method** | Cox regression; Entry at time of blood draw used for genotyping (baseline). Covariates come from baseline exam. Censoring at death or loss to follow-up | Conditional logistic regression. | Cox regression for case-cohort setting; Entry at time of blood draw used for genotyping (baseline). Covariates come from baseline exam. Censoring at death or loss to follow-up | Conditional logistic regression. | Cox regression; entry at randomization date; censoring at time of event, death or loss to follow-up; covariates from baseline questionnaire | Cox regression; Entry at time of blood draw used for genotyping (baseline). Covariates come from baseline exam. Censoring at death or loss to follow-up | Cox regression; entry at randomization date; censoring at time of event, death or loss to follow-up; covariates from baseline questionnaire |
| **Analysis software** | R version 2.10  ([www.r-project.org](http://www.r-project.org/)) | ProbABEL(http://mga.bionet.nsc.ru/~yurii/ABEL/) and SAS | R version 2.7  ([www.r-project.org](http://www.r-project.org/)) | ProbABEL(http://mga.bionet.nsc.ru/~yurii/ABEL/) and SAS 9.2 | ProbABEL(http://mga.bionet.nsc.ru/~yurii/ABEL/) | ProbABEL v. 0.1-3 | SAS 9.1, ProbABEL(http://mga.bionet.nsc.ru/~yurii/ABEL/), bash scripting |
