# Supplementary material for: Genome-Wide Association Study for Incident Myocardial Infarction and Coronary Heart Disease in Prospective Cohort Studies: The CHARGE Consortium
Source: PLoS One. 2016 Mar 7;11(3):e0144997. doi: 10.1371/journal.pone.0144997 (PMC4780701; doi:10.1371/journal.pone.0144997)
Supplement: S5 Table — (DOCX) [file pone.0144997.s008.docx]

### ****S5 Table - Genotyping/imputation/QC specifics of the studies in stage I****

|  | ***AGES*** | ***ARIC*** | ***CHS*** | ***FHS*** | ***The Rotterdam Study*** |
| --- | --- | --- | --- | --- | --- |
| **Genotyping platforms** | Illumina Human 370 CNV | Affymetrix 6.0 | Illumina HumanHap 370CNV | Affymetrix 250K Nsp, 250K Sty, 50K gene focused | Version 3 Illumina Infinium II HumanHap550 |
| **Genotyping center** | NIA | Broad Institute | Cedars-Sinai, Rotter + Taylor | Affymetrix | Erasmus MC |
| **Genotyping calling algorithm** | Illumina BeadStudio | BirdSeed | Illumina BeadStudio | BRLMM | Illumina BeadStudio |
| **Sample Call rate threshold** | > 95% | >95% | > 95% | > 97% | ≤ 90% |
| **Exclusion on race/ethnicity** | None | Non-European Americans | African-Americans not included | None | None |
| **Other Sample exclusions** | Sample failures, genotyped sex different from recorded sex, discordance with prior genotyping | Sample failures, genotyped sex different from recorded sex, discordance with prior genotyping, suspected first-degree relative of an included individual based on genome-wide genotype data, ) genetic outlier (as assessed by average Identity by State (IBS) | Sample failures, genotyped sex different from recorded sex, discordance with prior genotyping, | Sample failures, sex mismatch, excessive Mendelian errors (n=2), heterozygosity More than 5 SD from the mean (n=24) | Gender mismatch (n=36), heterozygosity excess autosomal heterozygosity > 0.336 (~FDR <0.1% [n=21]), outliers identified by the IBS clustering analysis > 3 standard deviations from population mean (n=102) IBS probabilities > 97% (n=129) |
| **SNP Call rate filter** | 97% | 90% | 97% | 97% | 90% |
| **SNP MAF threshold** | ≤1% | None | SNPs with variance dosage < 0.01 excluded (~MAF 0.05) | None | ≤1% (24977 SNPs) |
| **SNP HWE filter** | <1×10^-6^ | 10^-6^ (only for MAF >0.05) | <1×10^-5^ | <1×10^-6^ | <1×10^-6^ |
| **Other SNP filters.** | mismatch previous genotypes, remove A/T and G/C, not in  HapMap | SNPs whose genotype frequencies between 2 freezes differed by p<10^-6^/21,395 | No observed heterozygotes  Not present in HapMap reference panel, > 2 duplicate errors or Mendelian inconsistencies (for reference CEPH trios), heterozygote frequency = 0 | 45,361 SNPs (mishap P<1.0x10‾9), 4,857 SNPs (>100 Mendelian errors), 2 SNPs (due to strand issues upon merging data with HapMap), 13,394 SNPs not present on HapMap Phase 2. | None |
| **N SNPs for imputation** | 308,340 | 589,253 | 306,655 | 378,163 | 530,683 |
| **Imputation software** | MACH 1.0.16 | MACH 1.0 | BIMBAM v.0.99 | MACH 1.0.15 | MACH |
| **Imputation quality metrics** | R2 > 0.30 | None | variance dosage > 0.01 | None | None |
| **Imputation: reference panel** | HapMap release 22 CEU, Build 36 | HapMap release 22 CEU, Build 36 | HapMap release 22 CEU, Build 36 | HapMap release 22 CEU, build 36 | HapMap release 22 CEU, build 36 |
| **N SNPs used for imputation** | 308,340 | 589,253 | 306,665 |  | 530,683 |
| **N imputed SNPs for analysis** | 2,396,650 | 2,557,252 | 2,334,835 | 2,543,887 | 2,586,725 |

BIMBAM: <http://stephenslab.uchicago.edu/software.html>

MACH: <http://www.sph.umich.edu/csg/abecasis/MaCH/index.html>
