# Supplementary material for: Genome-Wide Association Study for Incident Myocardial Infarction and Coronary Heart Disease in Prospective Cohort Studies: The CHARGE Consortium
Source: PLoS One. 2016 Mar 7;11(3):e0144997. doi: 10.1371/journal.pone.0144997 (PMC4780701; doi:10.1371/journal.pone.0144997)
Supplement: S6 Table — (DOCX) [file pone.0144997.s009.docx]

### ****S6 Table - Genotyping/imputation/QC specifics of the studies in stage II****

|  | ***Health ABC*** | ***HPFS*** | ***MORGAM*** | ***NHS*** | ***PROSPER*** | ***SHIP*** | ***WGHS*** |
| --- | --- | --- | --- | --- | --- | --- | --- |
| **Genotyping platforms** | Illumina Human1M-Duo BeadChip | Affymetrix 6.0 | Sequenom MassARRAY | Affymetrix 6.0 | Illumina human 660-Quad beadchip | Affymetrix SNP Array 6.0 | Illumina HumanHap  300 DuoPlus |
| **Genotyping center** | Center for Inherited Disease Research (CIDR) | Merck/Rosetta | National Institute for Health and Welfare | Merck/Rosetta | Erasmus MC | Greifswald University, Affymetrix, Inc. | BWH/Amgen |
| **Genotyping calling algorithm** | Illumina BeadStudio 3.3.7 | Birdseed | Typer 4.0 | Birdseed | Illumina Beadstudio | Birdseed2 | Illumina BeadStudio 3.3 |
| **Sample Call rate threshold** | 95% | 95% | 95% | 95% | 98% | > 92% | 98% |
| **Exclusion on race/ethnicity** | African-Americans not included | Substantial similarity to non-European reference samples (either the HapMap YRI or CHB+JPT samples) were excluded (n=16) | none | Substantial similarity to non-European reference samples (either the HapMap YRI or CHB+JPT samples) were excluded (n=8) | Caucasians only | Caucasians only (verified by MDS) | Only verified self-report Europeans included |
| **Other Sample exclusions** | None | Sample failures, genotyped sex different from recorded sex, duplications | genotyping failures, genotyped sex different from recorded sex | sample failures, genotyped sex different from recorded sex, duplications | Sample failures, genotyped sex different from recorded seks, familial relationships | genotyped sex different from recorded sex, duplicates by estimated IBD | None |
| **SNP Call rate filter** | 90% | 97% | none | 97% | 97% | None | 90% |
| **SNP MAF threshold** | 1% | 2% | none | 2% | ≤1% | None | 1% |
| **SNP HWE filter** | 10^-6^ | 10^-4^ | <0.01 in >3/11 subcohorts | 10-4 | < 10^-6^ | None | < 10^-6^ |
| **Other SNP filters.** | None | None | none | None | None | None | None |
| **N SNPs for imputation** |  | 724,881 | none | 721,316 | 557,192 | 869,224 | 317186 |
| **Imputation software** | MACH 1.0.16 | MACH | NA | MACH | MACH 1.0.15 | IMPUTE v0.5.0 | MACH v1.0.15 |
| **Imputation quality metrics** | None | Quality score and Rsq | NA | Quality score and Rsq | None | None | None |
| **Imputation: reference panel** | HapMap CEU, release 22, Build 36 | HapMap CEU, release 22, Build 36 | NA | HapMap CEU, release 22, Build 36 | HapMap release 22 CEU, build 36 | HapMapII CEU, Build 36 | HapMap CEU, release 22, Build 36 |
| **Other SNP quality filters** | None | Rsq > 0.3 | NA | Rsq > 0.3 | None | None | None |
| **N SNPs used for imputation** | 958,777 autosomal | 724,881 | NA | 721,316 | 557,192 | 869,224 autosomal | 340,349 |
| **N imputed SNPs for analysis** | 2,543,887 | 2,543,887 * | NA | 2,543,887 * | 2,543,887 | 2,748,910 * | 2,608,508 |
| **Phenotype sample, N** | 1661 | 1330 | 6273** | 1420 | 5244 | 4308 | 23294 |
| **Exclusions, N** | None | 17 | NA | 11 | 1 | 425 | 0 |
| **Individuals for analysis, N** | 1661 | 1313 | 6018** | 1409 (sometimes less) | 5243 | 3883 | 23294 |

*Includes non-imputed SNPs present on AFffy 6.0 array but not in HapMap

** This refers to the full MORGAM case-cohort set, including end-points which were not used for this analysis

BIMBAM: <http://stephenslab.uchicago.edu/software.html>

MACH: <http://www.sph.umich.edu/csg/abecasis/MaCH/index.html>
