# Supplementary material for: Genome-Wide Association Study for Incident Myocardial Infarction and Coronary Heart Disease in Prospective Cohort Studies: The CHARGE Consortium
Source: PLoS One. 2016 Mar 7;11(3):e0144997. doi: 10.1371/journal.pone.0144997 (PMC4780701; doi:10.1371/journal.pone.0144997)
Supplement: S7 Table — (DOCX) [file pone.0144997.s010.docx]

### ****S7 Table - Basic description of the study population for the survival after MI analysis****

|  | ***AGES*** | ***ARIC*** | ***CHS*** | ***FHS*** | ***RS*** |
| --- | --- | --- | --- | --- | --- |
| **Number MI (deaths)** | 326 (101) | 978 (419) | 922 (798) | 165 (106) | 562 (404) |
| **Age at MI, years *** | 79.9 (6.1) | 62.0(6) | 83.1 (6.7) | 75.7 (12) | 76.5 (8) |
| **Women, %** | 53.40% | 34.80% | 53.5% | 41.80% | 59.4% |
| **Hypertension, %** | 86.20% | 52.90% | 71.8% | 75.90% | 54.8% |
| **Diabetes, %** | 14.40% | 26.00% | 23.5% | 28.90% | 10.6% |
| **Current smoker, %** | 14.10% | 28.70% | 7.7% | 19.50% | 22.1% |
| **Total cholesterol, mg/dL** | 224.6 (43.8) | 212(42.7) | 201.2 (39.6) | 197.2 (42.5) | 255 (46.4) |
| **HDL cholesterol, mg/dL** | 58.9 (15.7) | 42.2(13.2) | 51.0 (13.3) | 46.4 (15.5) | 54.1 (15.5) |
| **BMI, kg/m2 *** | 27.4 (4.3) | 28.3(5.0) | 26.5 (4.4) | 28.2 (4.8) | 29.1 (14.6) |
| **Survival time, years *** | 2.4 (1.8) | 7.6(5.9) | 3.4 (4.8) | 5.5 (4.8) | 5.5 (5.6) |
| **Survival time (min, 25th, Median, 75th, max))** | (0. 0.6, 2.3, 3.7, 7.1) | (0,2.3,7.0,11.9,22.2) | (0, 0, 0.39, 5.7, 21.5) | (0, 0.42, 4.7, 9.2, 17.5) | (0, 0.23, 3.8, 9.3, 22.3) |

* MAF of the coded allele in survivors / general population
