# Supplementary material for: Genome-Wide Association Study for Incident Myocardial Infarction and Coronary Heart Disease in Prospective Cohort Studies: The CHARGE Consortium
Source: PLoS One. 2016 Mar 7;11(3):e0144997. doi: 10.1371/journal.pone.0144997 (PMC4780701; doi:10.1371/journal.pone.0144997)
Supplement: S8 Table — (DOCX) [file pone.0144997.s011.docx]

### ****S8 Table - Basic description of the cohort studies in stage II****

| **Characteristic** | ***Health ABC*** | ***MORGAM*** | ***PROSPER/PHASE*** | ***SHIP*** | ***WGHS*** |
| --- | --- | --- | --- | --- | --- |
| **N** | 1661 | 3479* | 5243 | 3883 | 23294 |
| **Age, years** | 73.78 (2.8) | 52.08 (11.2) | 75.34 (3.4) | 49.0 (16.1) | 54.2 (7.1) |
| **Women, %** | 47 | 24 | 52 | 52 | 100 |
| **Hypertension, %** | 64 | 46 | 62 | 50 | 25 |
| **Diabetes, %** | 20 | 5 | 10 | 10 | 2.5 |
| **Current smoker, %** | 6 | 34 | 27 | 27 | 12 |
| **Total cholesterol, mg/dL** | 201 (38) | 224 (42) | 220 (35) | 223 (48) ** | 212 (42) |
| **HDL cholesterol, mg/dL** | 52 (16) | 50 (14) | 50 (14) | 56 (17) ** | 54 (15) |
| **Triglyceride, mg/dL** | 153.01 (88) | NA | 137 (63) | 163 (130) ** | 144 (92) |
| **BMI, kg/m2** | 26.6 (4.1) | 26.7 (4.2) | 26.8 (4.2) | 23.0 (4.1) | 25.9 (5.0) |
| **Prevalent CVD, %** | 26% | 0% | 44.5 | 0 | 0 |
| **Incident MI, N cases, average follow-up time** | 176, 5.46 (3.29) | 1114, 8.74 | 589, 3.10 (0.77) | 133, 9.34 (4.11) | 315, 13.7 |
| **Incident CHD, N cases, average follow-up time** | 456, 4.94 (3.17) | 1387, 8.71 | 589, 3.10 (0.77) | NA | 327, 13.7 |
| **Incident MI Age, years** | 78.8 (4.01) | 64.0 | 78.44 (3.40) | 67.9 (11.46) | 66.4 (8.1) |
| **Incident CHD Age, years** | 78.26 (3.98) | 63.6 | 78.44 (3.40) | NA | 66.8 (8.5) |

*This is the size of the case-cohort set used for incident CHD. The baseline characteristics in this column were weighted back to reflect the full cohort (excluding prevalent CVD).

** Not fasting
