# Supplementary material for: Genome-Wide Association Study for Incident Myocardial Infarction and Coronary Heart Disease in Prospective Cohort Studies: The CHARGE Consortium
Source: PLoS One. 2016 Mar 7;11(3):e0144997. doi: 10.1371/journal.pone.0144997 (PMC4780701; doi:10.1371/journal.pone.0144997)
Supplement: S9 Table — (DOCX) [file pone.0144997.s012.docx]

### ****S9 Table - Basic description of the case-control studies in stage II****

| **Characteristic** | ***HPFS*** | | ***NHS*** | |
| --- | --- | --- | --- | --- |
|  | ***Cases*** | ***Controls*** | ***Cases*** | ***Controls*** |
| **N** | 425 | 878 | 464 | 945 |
| **Age, years (SD)** | 64.5 (8.6) | 64.2 (8.5) | 60.2 (6.3) | 59.8 (6.3) |
| **Women, %** | 0 | 0 | 100 | 100 |
| **Hypertension, %** | 37.2 | 29.0 | 50.2 | 27.3 |
| **Diabetes, %** | 9.0 | 3.8 | 14.4 | 6.24 |
| **Current smoker, %** | 9.7 | 8.7 | 27.8 | 26.1 |
| **Total cholesterol, mg/dL** | 211 (39) | 203 (37) | 234 (41) | 226 (41) |
| **HDL cholesterol, mg/dL** | 43 (11) | 46 (13) | 52 (15) | 60 (17) |
| **Triglyceride, mg/dL** | 163 (111) | 134 (199) | 146 (86) | 118 (64) |
| **BMI, kg/m2** | 26.0 (3.2) | 25.6 (3.3) | 26.0 (6.6) | 24.5 (5.8) |
