# Supplementary material for: Genome-Wide Association Study for Incident Myocardial Infarction and Coronary Heart Disease in Prospective Cohort Studies: The CHARGE Consortium
Source: PLoS One. 2016 Mar 7;11(3):e0144997. doi: 10.1371/journal.pone.0144997 (PMC4780701; doi:10.1371/journal.pone.0144997)
Supplement: S10 Table — (DOCX) [file pone.0144997.s013.docx]

### ****S10 Table - Association of the SNPs taken to stage II with MI****

| **SNPID** | **Chromosome** | **Position** | **Coded allele** | **Non-coded allele** | **Stage II** | | **Combined** | | **Closest Gene** |
| --- | --- | --- | --- | --- | --- | --- | --- | --- | --- |
|  |  |  |  |  | **OR (95%CI)** | **P-value** | **OR (95%CI)** | **P-value** |  |
| rs1570488 | 6 | 16791909 | T | C | 0.93 ( 0.78 - 1.11 ) | 0.415 | 0.84 ( 0.77 - 0.91 ) | 7.0×10^-5^ | ATXN1 |
| rs2299063 | 6 | 16757620 | A | C | 1.08 ( 0.94 - 1.23 ) | 0.289 | 0.88 ( 0.81 - 0.95 ) | 1.6×10^-3^ | ATXN1 |
| rs7760476 | 6 | 16781608 | A | G | 0.99 ( 0.85 - 1.15 ) | 0.882 | 1.15 ( 1.06 - 1.25 ) | 9.4×10^-4^ | ATXN1 |
| rs9297015 | 6 | 16744990 | A | T | 1.09 ( 0.94 - 1.26 ) | 0.259 | 0.89 ( 0.82 - 0.96 ) | 4.6×10^-3^ | ATXN1 |
| rs10164679 | 2 | 215375393 | T | C | 1.02 ( 0.87 - 1.19 ) | 0.803 | 0.87 ( 0.81 - 0.94 ) | 1.8×10^-4^ | BARD1 |
| rs10498023 | 2 | 215369955 | T | C | 1.13 ( 0.78 - 1.64 ) | 0.504 | 0.76 ( 0.65 - 0.9 ) | 9.9×10^-4^ | BARD1 |
| rs7591615 | 2 | 215372636 | T | C | 0.89 ( 0.78 - 1.02 ) | 0.091 | 1.11 ( 1.03 - 1.19 ) | 3.8×10^-3^ | BARD1 |
| rs9973844 | 2 | 215376394 | A | G | 0.98 ( 0.84 - 1.14 ) | 0.802 | 1.15 ( 1.07 - 1.23 ) | 1.7×10^-4^ | BARD1 |
| rs10922855 | 1 | 90640761 | T | G | 0.95 ( 0.79 - 1.16 ) | 0.629 | 1.14 ( 1.05 - 1.25 ) | 3.2×10^-3^ | BARHL2 |
| rs10922857 | 1 | 90654688 | T | C | 0.95 ( 0.8 - 1.12 ) | 0.523 | 1.13 ( 1.03 - 1.23 ) | 6.5×10^-3^ | BARHL2 |
| rs12031583 | 1 | 90699057 | A | G | 1.02 ( 0.84 - 1.24 ) | 0.84 | 0.87 ( 0.79 - 0.95 ) | 1.7×10^-3^ | BARHL2 |
| rs17131045 | 1 | 90674871 | A | T | 0.95 ( 0.78 - 1.15 ) | 0.606 | 1.14 ( 1.05 - 1.25 ) | 3.1×10^-3^ | BARHL2 |
| rs16841958 | 3 | 101365705 | T | C | 0.77 ( 0.49 - 1.21 ) | 0.251 | 0.63 ( 0.51 - 0.78 ) | 1.7×10^-5^ | C3orf26 |
| rs17777478 | 3 | 100868388 | A | T | 0.87 ( 0.61 - 1.24 ) | 0.451 | 0.65 ( 0.53 - 0.79 ) | 1.6×10^-5^ | COL8A1 |
| rs10509258 | 10 | 67453443 | A | G | 0.98 ( 0.87 - 1.10 ) | 0.705 | 1.09 ( 1.02 - 1.16 ) | 1.0×10^-2^ | CTNNA3 |
| rs10740220 | 10 | 67460152 | T | G | 1.08 ( 0.94 - 1.24 ) | 0.26 | 0.91 ( 0.85 - 0.98 ) | 1.5×10^-2^ | CTNNA3 |
| rs2893978 | 10 | 67452838 | T | C | 1.05 ( 0.91 - 1.21 ) | 0.527 | 0.91 ( 0.85 - 0.98 ) | 1.1×10^-2^ | CTNNA3 |
| rs6480128 | 10 | 67453139 | T | C | 1.03 ( 0.91 - 1.16 ) | 0.649 | 0.92 ( 0.86 - 0.98 ) | 1.2×10^-2^ | CTNNA3 |
| rs2041407 | 7 | 14827626 | T | C | 0.99 ( 0.88 - 1.10 ) | 0.824 | 0.9 ( 0.85 - 0.96 ) | 1.3×10^-3^ | DGKB |
| rs2041408 | 7 | 14828413 | A | G | 1.02 ( 0.89 - 1.16 ) | 0.79 | 0.91 ( 0.85 - 0.97 ) | 2.4×10^-3^ | DGKB |
| rs4721377 | 7 | 14821587 | T | C | 0.98 ( 0.87 - 1.10 ) | 0.73 | 0.9 ( 0.84 - 0.96 ) | 1.2×10^-3^ | DGKB |
| rs6968756 | 7 | 14820434 | T | C | 1.00 ( 0.89 - 1.12 ) | 0.936 | 0.9 ( 0.85 - 0.96 ) | 1.7×10^-3^ | DGKB |
| rs1489719 | 3 | 101332824 | A | T | 1.17 ( 0.81 - 1.70 ) | 0.408 | 1.52 ( 1.24 - 1.87 ) | 5.3×10^-5^ | FILIP1L |
| rs16841920 | 3 | 101266610 | T | C | 0.86 ( 0.59 - 1.25 ) | 0.417 | 0.64 ( 0.52 - 0.78 ) | 2.0×10^-5^ | FILIP1L |
| rs16941787 | 16 | 85234040 | T | G | 0.97 ( 0.75 - 1.26 ) | 0.813 | 1.26 ( 1.11 - 1.42 ) | 2.6×10^-4^ | FOXL1 |
| rs16941789 | 16 | 85234265 | T | C | 1.03 ( 0.80 - 1.34 ) | 0.802 | 0.8 ( 0.71 - 0.9 ) | 2.7×10^-4^ | FOXL1 |
| rs4843416 | 16 | 85242920 | A | G | 0.71 ( 0.51 - 0.99 ) | 0.044 | 0.7 ( 0.6 - 0.82 ) | 6.5×10^-6^ | FOXL1 |
| rs9923194 | 16 | 85225483 | T | C | 0.96 ( 0.63 - 1.45 ) | 0.84 | 0.63 ( 0.51 - 0.79 ) | 4.3×10^-5^ | FOXL1 |
| rs10454553 | 13 | 90561140 | A | G | 1.07 ( 0.90 - 1.28 ) | 0.451 | 0.85 ( 0.77 - 0.95 ) | 4.8×10^-3^ | GPC5 |
| rs1380336 | 13 | 90531168 | T | C | 0.86 (0.72 - 1.04 ) | 0.119 | 1.11 ( 0.98 - 1.25 ) | 1.1×10^-1^ | GPC5 |
| rs16945166 | 13 | 90543572 | A | G | 1.12 ( 0.92 - 1.36 ) | 0.264 | 0.85 ( 0.76 - 0.96 ) | 6.5×10^-3^ | GPC5 |
| rs16945184 | 13 | 90553200 | T | C | 0.91 ( 0.74 - 1.11 ) | 0.336 | 0.81 ( 0.73 - 0.9 ) | 1.1×10^-4^ | GPC5 |
| rs10241906 | 7 | 46384028 | T | C | 0.96 ( 0.82 - 1.12 ) | 0.597 | 1.09 ( 1.01 - 1.18 ) | 2.2×10^-2^ | IGFBP3 |
| rs1551837 | 7 | 46400581 | A | G | 0.96 ( 0.82 - 1.12 ) | 0.606 | 1.09 ( 1.01 - 1.17 ) | 2.7×10^-2^ | IGFBP3 |
| rs7789988 | 7 | 46381846 | A | C | 1.02 ( 0.90 - 1.16 ) | 0.774 | 0.92 ( 0.86 - 0.99 ) | 2.5×10^-2^ | IGFBP3 |
| rs7794677 | 7 | 46368625 | T | C | 0.97 ( 0.86 - 1.11 ) | 0.688 | 1.1 ( 1.02 - 1.18 ) | 1.5×10^-2^ | IGFBP3 |
| rs13192150 | 6 | 40495722 | T | C | 0.88 ( 0.73 - 1.06 ) | 0.191 | 0.87 ( 0.79 - 0.95 ) | 2.8×10^-3^ | LRFN2 |
| rs2916260 | 6 | 40490689 | T | C | 0.97 ( 0.83 - 1.12 ) | 0.664 | 0.89 ( 0.83 - 0.97 ) | 7.2×10^-3^ | LRFN2 |
| rs6925172 | 6 | 40499542 | T | C | 0.89 ( 0.74 - 1.07 ) | 0.214 | 0.87 ( 0.79 - 0.95 ) | 2.8×10^-3^ | LRFN2 |
| rs892367 | 6 | 40477590 | T | C | 0.98 ( 0.83 - 1.15 ) | 0.782 | 0.89 ( 0.81 - 0.98 ) | 1.6×10^-2^ | LRFN2 |
| rs10434298 | 4 | 183859895 | T | C | 1.02 ( 0.79 - 1.32 ) | 0.892 | 1.31 ( 1.16 - 1.48 ) | 7.7×10^-6^ | ODZ3 |
| rs4398556 | 4 | 183857042 | A | G | 1.02 ( 0.79 - 1.32 ) | 0.871 | 1.31 ( 1.16 - 1.48 ) | 8.7×10^-6^ | ODZ3 |
| rs6552596 | 4 | 183861195 | C | G | 1.03 ( 0.76 - 1.39 ) | 0.869 | 0.76 ( 0.67 - 0.86 ) | 9.7×10^-6^ | ODZ3 |
| rs7692395 | 4 | 183861638 | T | G | 1.03 ( 0.76 - 1.39 ) | 0.865 | 0.76 ( 0.67 - 0.86 ) | 1.0×10^-5^ | ODZ3 |
| rs10875195 | 1 | 99138510 | T | C | 0.95 ( 0.85 - 1.07 ) | 0.428 | 0.88 ( 0.83 - 0.93 ) | 3.1×10^-5^ | PAP2D |
| rs1383832 | 1 | 99140432 | A | G | 0.98 ( 0.85 - 1.12 ) | 0.718 | 0.89 ( 0.83 - 0.94 ) | 2.2×10^-4^ | PAP2D |
| rs1871786 | 1 | 99138751 | T | C | 1.02 ( 0.89 - 1.16 ) | 0.81 | 1.13 ( 1.06 - 1.2 ) | 2.9×10^-4^ | PAP2D |
| rs986080 | 1 | 99100295 | T | C | 1.04 ( 0.92 - 1.25 ) | 0.561 | 1.14 (1.06 - 1.22 ) | 8.1×10^-5^ | PAP2D |
| rs6941513 | 6 | 163950790 | A | G | 0.86 ( 0.77 - 0.96 ) | 0.009 | 0.83 ( 0.78 - 0.89 ) | 6.2×10^-9^ | QKI |
| rs7745161 | 6 | 163949460 | C | G | 0.86 ( 0.77 - 0.97 ) | 0.012 | 0.83 ( 0.78 - 0.89 ) | 1.3×10^-8^ | QKI |
| rs7751144 | 6 | 163943627 | A | G | 0.86 ( 0.75 - 0.99 ) | 0.034 | 0.84 ( 0.79 - 0.9 ) | 3.1×10^-7^ | QKI |
| rs7756185 | 6 | 163951611 | T | G | 1.19 ( 1.06 - 1.33 ) | 0.003 | 1.2 ( 1.13 - 1.28 ) | 1.4×10^-8^ | QKI |
| rs356168 | 4 | 90893454 | A | G | 0.93 ( 0.81 - 1.06 ) | 0.261 | 1.09 ( 1.02 - 1.16 ) | 1.1×10^-2^ | SNCA |
| rs356200 | 4 | 90887637 | T | G | 0.92 ( 0.83 - 1.03 ) | 0.136 | 1.06 (1.00 - 1.14 ) | 3.4×10^-2^ | SNCA |
| rs356204 | 4 | 90882565 | T | C | 1.08 ( 0.94 - 1.23 ) | 0.269 | 0.92 ( 0.86 - 0.98 ) | 1.1×10^-2^ | SNCA |
| rs356228 | 4 | 90826149 | C | G | 1.07 ( 0.95 - 1.2 ) | 0.287 | 0.91 ( 0.86 - 0.97 ) | 4.8×10^-3^ | SNCA |
| rs17707058 | 17 | 44195780 | T | C | 0.95 ( 0.85 - 1.07 ) | 0.39 | 0.88 ( 0.83 - 0.93 ) | 3.4×10^-5^ | TTLL6 |
| rs4793987 | 17 | 44210836 | T | C | 0.98 ( 0.86 - 1.12 ) | 0.772 | 0.88 ( 0.82 - 0.94 ) | 6.9×10^-5^ | TTLL6 |
| rs953258 | 17 | 44196992 | T | C | 1.04 ( 0.93 - 1.12 ) | 0.474 | 1.15 ( 1.08 - 1.22 ) | 8.6×10^-5^ | TTLL6 |
| rs6504582 | 17 | 44260055 | A | G | 1.03 ( 0.92 - 1.15 ) | 0.649 | 1.15 ( 1.08 - 1.22 ) | 1.8×10^-5^ | CALCOCO2 |
