# Supplementary material for: Genome-Wide Association Study for Incident Myocardial Infarction and Coronary Heart Disease in Prospective Cohort Studies: The CHARGE Consortium
Source: PLoS One. 2016 Mar 7;11(3):e0144997. doi: 10.1371/journal.pone.0144997 (PMC4780701; doi:10.1371/journal.pone.0144997)
Supplement: S11 Table — (DOCX) [file pone.0144997.s014.docx]

### ****S11 Table – Association of the SNPs taken to stage II with CHD****

| **SNPID** | **Chromosome** | **Position** | **Coded allele** | **Non-coded allele** | **Stage II** | | **Combined** | | **Closest Gene** |
| --- | --- | --- | --- | --- | --- | --- | --- | --- | --- |
|  |  |  |  |  | **OR (95%CI)** | **P-value** | **OR (95%CI)** | **P-value** |  |
| rs1570488 | 6 | 16791909 | T | C | 1.06 ( 0.96 - 1.17 ) | 0.245 | 0.92 ( 0.87 - 0.98 ) | 1.2×10^-2^ | ATXN1 |
| rs2299063 | 6 | 16757620 | A | C | 1.06 ( 0.99 - 1.15 ) | 0.091 | 1.05 ( 0.99 - 1.11 ) | 7.7×10^-2^ | ATXN1 |
| rs7760476 | 6 | 16781608 | A | G | 0.93 ( 0.86 - 1.01 ) | 0.082 | 1.05 ( 0.99 - 1.11 ) | 9.8×10^-2^ | ATXN1 |
| rs9297015 | 6 | 16744990 | A | T | 1.08 ( 1.00 - 1.17 ) | 0.065 | 0.96 ( 0.90 - 1.01 ) | 1.2×10^-1^ | ATXN1 |
| rs10164679 | 2 | 215375393 | T | C | 0.95 ( 0.87 - 1.03 ) | 0.204 | 0.91 ( 0.86 - 0.96 ) | 3.0×10^-4^ | BARD1 |
| rs10498023 | 2 | 215369955 | T | C | 1.06 ( 0.89 - 1.27 ) | 0.517 | 0.91 ( 0.81 - 1.03 ) | 1.3×10^-1^ | BARD1 |
| rs7591615 | 2 | 215372636 | T | C | 0.99 ( 0.92 - 1.07 ) | 0.831 | 1.06 ( 1.01 - 1.12 ) | 1.7×10^-2^ | BARD1 |
| rs9973844 | 2 | 215376394 | A | G | 1.06 ( 0.97 - 1.16 ) | 0.199 | 1.10 ( 1.05 - 1.17 ) | 2.9×10^-4^ | BARD1 |
| rs10922855 | 1 | 90640761 | T | G | 0.90 ( 0.80 - 1.01 ) | 0.075 | 1.10 ( 1.03 - 1.18 ) | 4.6×10^-3^ | BARHL2 |
| rs10922857 | 1 | 90654688 | T | C | 0.95 ( 0.87 - 1.05 ) | 0.304 | 1.10 ( 1.03 - 1.17 ) | 4.0×10^-3^ | BARHL2 |
| rs12031583 | 1 | 90699057 | A | G | 1.12 ( 1.00 - 1.26 ) | 0.053 | 0.91 ( 0.85 - 0.98 ) | 7.8×10^-3^ | BARHL2 |
| rs17131045 | 1 | 90674871 | A | T | 0.90 ( 0.80 – 1.00 ) | 0.06 | 1.10 ( 1.02 - 1.17 ) | 7.5×10^-3^ | BARHL2 |
| rs10509258 | 10 | 67453443 | A | G | 1.02 ( 0.96 - 1.10 ) | 0.496 | 0.92 ( 0.88 - 0.96 ) | 3.0×10^-4^ | CTNNA3 |
| rs10740220 | 10 | 67460152 | T | A | 0.99 ( 0.92 - 1.06 ) | 0.74 | 0.90 ( 0.86 - 0.95 ) | 1.8×10^-4^ | CTNNA3 |
| rs2893978 | 10 | 67452838 | T | C | 0.99 ( 0.91 - 1.08 ) | 0.824 | 0.91 ( 0.87 - 0.96 ) | 4.3×10^-4^ | CTNNA3 |
| rs6480128 | 10 | 67453139 | T | G | 0.98 ( 0.92 - 1.05 ) | 0.575 | 0.92 ( 0.88 - 0.96 ) | 4.0×10^-4^ | CTNNA3 |
| rs2041407 | 7 | 14827626 | T | C | 0.98 ( 0.92 - 1.05 ) | 0.59 | 0.95 ( 0.91 - 0.99 ) | 1.4×10^-2^ | DGKB |
| rs2041408 | 7 | 14828413 | A | G | 1.00 ( 0.93 - 1.08 ) | 0.996 | 0.96 ( 0.91 – 1.00 ) | 5.8×10^-2^ | DGKB |
| rs4721377 | 7 | 14821587 | T | C | 0.97 ( 0.91 - 1.04 ) | 0.412 | 0.94 ( 0.90 - 0.99 ) | 1.2×10^-2^ | DGKB |
| rs6968756 | 7 | 14820434 | T | C | 0.99 ( 0.93 - 1.06 ) | 0.794 | 0.95 ( 0.91 - 0.99 ) | 2.1×10^-2^ | DGKB |
| rs1489719 | 3 | 101332824 | A | G | 1.14 ( 0.94 - 1.37 ) | 0.177 | 1.27 ( 1.10 - 1.45 ) | 8.4×10^-4^ | FILIP1L |
| rs16841920 | 3 | 101266610 | T | C | 0.84 ( 0.69 - 1.02 ) | 0.071 | 0.75 ( 0.66 - 0.87 ) | 1.0×10^-4^ | FILIP1L |
| rs16841958 | 3 | 101365705 | T | A | 0.83 ( 0.65 - 1.07 ) | 0.148 | 0.75 ( 0.64 - 0.88 ) | 3.4×10^-4^ | C3orf26 |
| rs17777478 | 3 | 100868388 | A | G | 0.82 ( 0.68 - 0.97 ) | 0.024 | 0.75 ( 0.65 - 0.85 ) | 2.0×10^-5^ | COL8A1 |
| rs16941787 | 16 | 85234040 | T | G | 0.99 ( 0.86 - 1.14 ) | 0.858 | 1.14 ( 1.04 - 1.25 ) | 4.2×10^-3^ | FOXL1 |
| rs16941789 | 16 | 85234265 | T | C | 1.01 ( 0.88 - 1.16 ) | 0.888 | 0.88 ( 0.80 - 0.96 ) | 3.8×10^-3^ | FOXL1 |
| rs4843416 | 16 | 85242920 | A | G | 0.97 ( 0.8 - 1.18 ) | 0.784 | 0.83 ( 0.74 - 0.94 ) | 2.4×10^-3^ | FOXL1 |
| rs9923194 | 16 | 85225483 | T | C | 1.03 ( 0.82 - 1.29 ) | 0.793 | 0.82 ( 0.70 - 0.96 ) | 1.4×10^-2^ | FOXL1 |
| rs10454553 | 13 | 90561140 | A | G | 1.00 ( 0.91 - 1.10 ) | 0.958 | 0.89 ( 0.82 - 0.95 ) | 1.3×10^-3^ | GPC5 |
| rs1380336 | 13 | 90531168 | T | C | 1.00 ( 0.90 - 1.11 ) | 0.98 | 0.89 ( 0.82 - 0.97 ) | 4.7×10^-3^ | GPC5 |
| rs16945166 | 13 | 90543572 | A | G | 0.98 ( 0.88 - 1.09 ) | 0.687 | 0.86 ( 0.80 - 0.93 ) | 2.2×10^-4^ | GPC5 |
| rs16945184 | 13 | 90553200 | T | C | 0.90 ( 0.79 - 1.02 ) | 0.086 | 0.83 ( 0.77 - 0.90 ) | 5.8×10^-6^ | GPC5 |
| rs10241906 | 7 | 46384028 | T | C | 1.02 ( 0.93 - 1.12 ) | 0.634 | 1.11 ( 1.05 - 1.18 ) | 2.1×10^-4^ | IGFBP3 |
| rs1551837 | 7 | 46400581 | A | G | 1.03 ( 0.94 - 1.12 ) | 0.576 | 1.11 ( 1.05 - 1.18 ) | 2.3×10^-4^ | IGFBP3 |
| rs7789988 | 7 | 46381846 | A | C | 0.99 ( 0.92 - 1.06 ) | 0.679 | 0.92 ( 0.87 - 0.96 ) | 6.1×10^-4^ | IGFBP3 |
| rs7794677 | 7 | 46368625 | T | C | 1.02 ( 0.95 - 1.09 ) | 0.643 | 1.10 ( 1.04 - 1.16 ) | 3.3×10^-4^ | IGFBP3 |
| rs13192150 | 6 | 40495722 | T | C | 0.89 ( 0.80 – 1.00 ) | 0.041 | 0.85 ( 0.80 - 0.91 ) | 4.1×10^-6^ | LRFN2 |
| rs2916260 | 6 | 40490689 | T | C | 0.92 ( 0.85 - 1.00 ) | 0.045 | 1.15 ( 1.09 - 1.22 ) | 1.8×10^-6^ | LRFN2 |
| rs6925172 | 6 | 40499542 | T | C | 0.89 ( 0.80 - 0.99 ) | 0.038 | 0.85 ( 0.79 - 0.91 ) | 3.3×10^-6^ | LRFN2 |
| rs892367 | 6 | 40477590 | T | C | 0.95 ( 0.87 - 1.05 ) | 0.348 | 1.14 ( 1.07 - 1.21 ) | 1.1×10^-4^ | LRFN2 |
| rs10434298 | 4 | 183859895 | T | C | 1.04 ( 0.90 - 1.21 ) | 0.565 | 1.15 ( 1.05 - 1.26 ) | 3.3×10^-3^ | ODZ3 |
| rs4398556 | 4 | 183857042 | A | G | 1.05 ( 0.91 - 1.22 ) | 0.488 | 1.15 ( 1.05 - 1.26 ) | 2.9×10^-3^ | ODZ3 |
| rs6552596 | 4 | 183861195 | C | G | 0.94 ( 0.80 - 1.11 ) | 0.444 | 0.86 ( 0.78 - 0.95 ) | 2.0×10^-3^ | ODZ3 |
| rs7692395 | 4 | 183861638 | T | G | 0.94 ( 0.80 - 1.10 ) | 0.441 | 0.86 ( 0.78 - 0.95 ) | 2.0×10^-3^ | ODZ3 |
| rs10875195 | 1 | 99138510 | T | C | 1.03 ( 0.96 - 1.09 ) | 0.438 | 0.93 ( 0.89 - 0.97 ) | 6.4×10^-4^ | PAP2D |
| rs1383832 | 1 | 99140432 | A | G | 1.01 ( 0.93 - 1.09 ) | 0.811 | 0.91 ( 0.87 - 0.95 ) | 1.2×10^-4^ | PAP2D |
| rs1871786 | 1 | 99138751 | T | C | 0.97 ( 0.90 - 1.05 ) | 0.48 | 1.09 ( 1.04 - 1.14 ) | 4.6×10^-4^ | PAP2D |
| rs986080 | 1 | 99100295 | T | C | 0.97 ( 0.91 - 1.04 ) | 0.392 | 0.93 ( 0.89 - 0.97 ) | 1.1×10^-3^ | PAP2D |
| rs6941513 | 6 | 163950790 | A | G | 0.94 ( 0.89 - 1.01 ) | 0.076 | 0.91 ( 0.87 - 0.95 ) | 3.5×10^-5^ | QKI |
| rs7745161 | 6 | 163949460 | C | G | 0.96 ( 0.90 - 1.02 ) | 0.202 | 0.92 ( 0.88 - 0.96 ) | 1.5×10^-4^ | QKI |
| rs7751144 | 6 | 163943627 | A | G | 0.94 ( 0.87 - 1.02 ) | 0.142 | 0.91 ( 0.87 - 0.96 ) | 2.3×10^-4^ | QKI |
| rs7756185 | 6 | 163951611 | T | G | 1.08 ( 1.01 - 1.15 ) | 0.022 | 1.11 ( 1.07 - 1.17 ) | 2.0×10^-6^ | QKI |
| rs356168 | 4 | 90893454 | A | G | 1.08 ( 1.00 - 1.16 ) | 0.056 | 1.10 ( 1.05 - 1.16 ) | 3.2×10^-5^ | SNCA |
| rs356200 | 4 | 90887637 | T | G | 1.04 ( 0.98 - 1.10 ) | 0.241 | 0.93 ( 0.89 - 0.97 ) | 4.2×10^-4^ | SNCA |
| rs356204 | 4 | 90882565 | T | C | 0.93 ( 0.86 – 1.00 ) | 0.056 | 0.91 ( 0.86 - 0.95 ) | 3.4×10^-5^ | SNCA |
| rs356228 | 4 | 90826149 | C | G | 0.98 ( 0.92 - 1.04 ) | 0.495 | 0.92 ( 0.88 - 0.96 ) | 1.7×10^-4^ | SNCA |
| rs17707058 | 17 | 44195780 | T | C | 0.98 ( 0.92 - 1.04 ) | 0.488 | 0.93 ( 0.89 - 0.97 ) | 1.8×10^-3^ | TTLL6 |
| rs4793987 | 17 | 44210836 | T | C | 0.98 ( 0.91 - 1.06 ) | 0.629 | 0.93 ( 0.88 - 0.97 ) | 2.2×10^-3^ | TTLL6 |
| rs953258 | 17 | 44196992 | T | C | 1.02 ( 0.96 - 1.09 ) | 0.489 | 0.94 ( 0.89 - 0.98 ) | 5.3×10^-3^ | TTLL6 |
| rs6504582 | 17 | 44260055 | A | G | 1.00 ( 0.94 - 1.07 ) | 0.9 | 1.07 ( 1.03 - 1.12 ) | 2.3×10^-3^ | CALCOCO2 |
